# Multi-agent meta-reinforcement learning based on contrastive predictive coding—For rapid adaptive traffic control after emergencies

**DOI:** 10.1371/journal.pone.0357779

**Published:** 2026-09-08

**Authors:** Wei Jiang, Xianwei Huang, Genjie Wang, Ruting Li, Shanshan Tang

**Affiliations:** 1 Faculty of Intelligent Transportation, Anhui Sanlian University, Hefei, Anhui, China; 2 Faculty of Engineering, Anhui Sanlian University, Hefei, Anhui, China; Southwest Jiaotong University, CHINA

## Abstract

Emergency signal control has to clear emergency vehicles through intersections without creating excessive delay for ordinary traffic. Many existing controllers still depend on manually designed traffic features, and unfamiliar emergency patterns often require additional training before the control policy can be adjusted reliably. For this setting, a multi-agent meta-reinforcement learning framework is developed using Contrastive Predictive Coding (CPC) for traffic state representation. CPC compresses high-dimensional traffic sequences into compact representations that are subsequently used by intersection agents during few-sample policy adaptation. Agent communication is weighted by both distance and transmission delay, while hierarchical emergency priority control adjusts signal decisions according to event severity. Tests on the iTETRIS simulation platform with the CityFlow Emergency dataset produced an emergency response time of 25.3 s, an average regular vehicle delay of 80.5 s per vehicle, and a 98.2% emergency success rate in high-priority scenarios. In the ablation experiments, removing CPC caused the largest single performance loss, whereas the all-module configuration showed a 44.3% contribution to response-time reduction in the contribution analysis. Overall, the framework maintained emergency vehicle priority while keeping the effect on regular traffic within a comparatively limited range under the evaluated emergency conditions.

## 1. Introduction

Managing emergency vehicle passage has become more difficult as urban traffic networks grow denser and more interconnected, particularly when signal priority must be provided without disrupting routine traffic. Delayed access for emergency medical vehicles can reduce rescue efficiency, and incidents may also trigger congestion around affected intersections. Previous studies report that each additional minute of emergency medical service delay may raise mortality risk by 7%, underscoring the operational consequences of slow response [1]. Most existing signal-control schemes still rely on fixed-time or semi-adaptive rules. When an unexpected event occurs, these schemes may react too slowly and coordinate poorly with neighboring intersections, which can delay emergency vehicles and disturb surrounding traffic [2,3]. Emergency traffic control therefore requires methods that can recognize changing conditions quickly, revise control decisions in real time, and give emergency vehicles priority without causing excessive deterioration in regular traffic flow.

Most studies on traffic control focus on routine congestion. Emergency situations are more difficult because the control policy may need to change quickly when an unfamiliar event occurs. Conventional approaches often depend on manually selected indicators, such as vehicle density and delay. These variables may not fully capture the spatiotemporal changes contained in high-dimensional traffic observations [4]. Contrastive Predictive Coding (CPC) has been widely used for representation learning in image and speech tasks, but its application to traffic sequences, including vehicle trajectories and changes in intersection flow, remains limited [5]. Its performance in representing rapidly changing traffic states during emergencies has not been examined in sufficient detail. Multi-Agent Reinforcement Learning (MARL) can coordinate control among several intersections. Many MARL methods still require a large number of interaction samples when adapting policies to new environments, which limits their use in emergency situations where decisions must be made quickly. Current studies also seldom connect emergency event classification directly with the decision process, making it difficult for one control policy to respond differently to different types of emergencies [6].

Adaptive traffic control research has increasingly considered representation learning and cooperative decision-making strategies for handling complex traffic dynamics. Multi-agent reinforcement learning approaches have introduced attention-based feature aggregation and distributed coordination strategies to improve traffic signal optimization under dynamic environments [7,8]. However, existing methods mainly emphasize coordination efficiency or large-scale control performance, whereas rapid adaptation to previously unseen emergency scenarios remains less explored. Efficient traffic state representation and adaptive policy optimization therefore need to be jointly considered for emergency conditions with limited interaction data.

This study develops a multi-agent meta-reinforcement learning framework that integrates contrastive predictive coding for rapid adaptive traffic control after emergencies. A CPC-based traffic state representation model is constructed to extract low-dimensional semantic features through temporal prediction tasks, reducing redundant information involved in the decision-making process. A multi-agent meta-reinforcement learning mechanism is then developed to exploit the few-shot adaptation capability of meta-learning, allowing intersection controller agents to adjust their strategies under emergency scenarios. In addition, a hierarchical adaptive control strategy is introduced based on emergency severity levels to balance emergency vehicle priority and regular traffic stability. The framework provides an adaptive solution for traffic signal control under diverse emergency scenarios.

**Fig 1:**
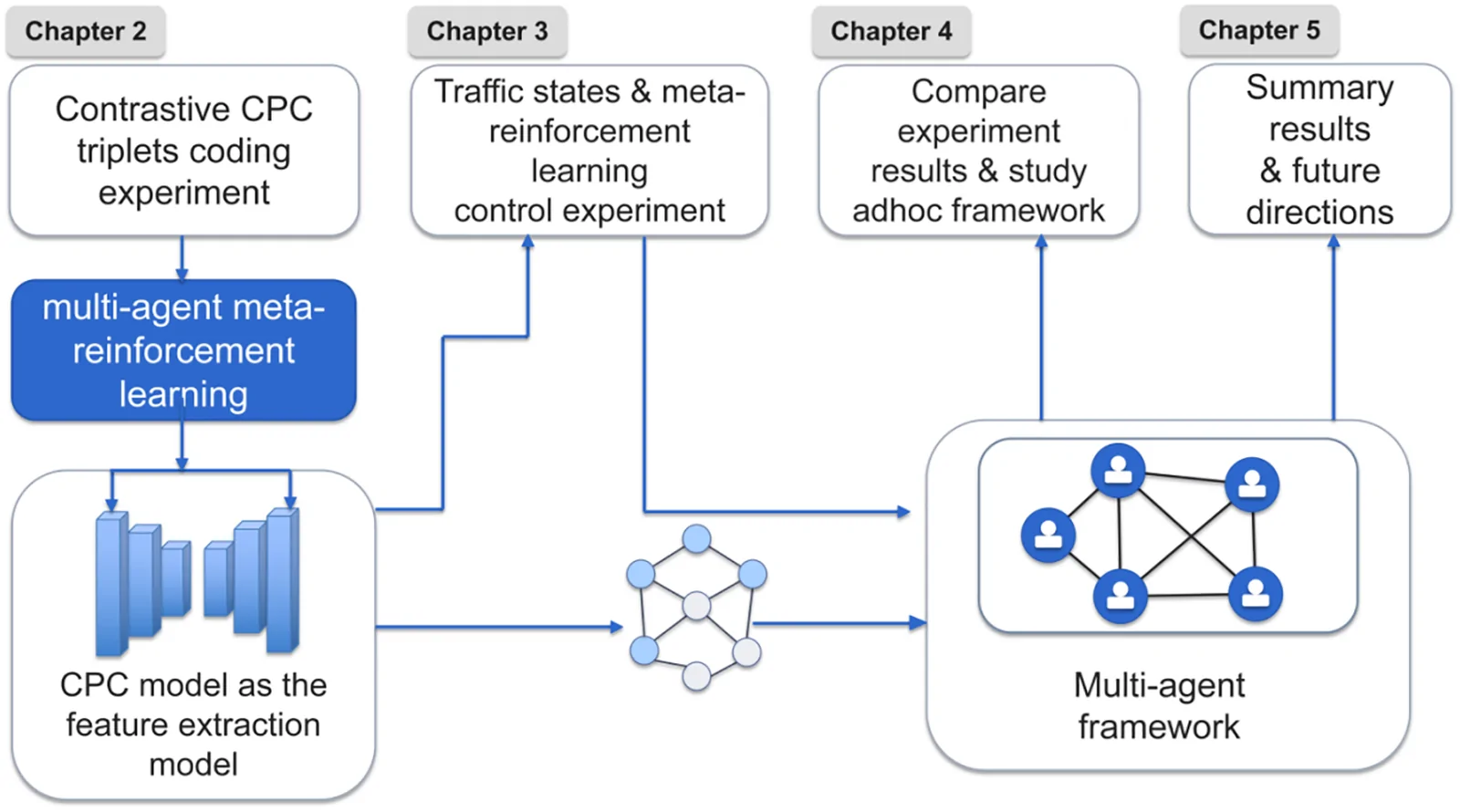
Article structure.

The overall framework of this study is organized around the proposed objectives, as shown in Fig 1.

Contributions and highlights of the article:

- The framework integrates multi-agent reinforcement learning with Contrastive Predictive Coding (CPC) to improve adaptive traffic control under emergency scenarios.
- Experimental results show that the proposed method reduces emergency response time by 15.9% and decreases regular vehicle delay by 9.2%.
- The proposed framework incorporates CPC-based representation learning, meta-learning adaptation, and multi-agent coordination, with the contribution analysis showing a 44.3% response-time reduction contribution when all modules are jointly applied.

The paper contains six sections. Section 2 introduces CPC, multi-agent meta-reinforcement learning, and the main requirements of emergency traffic control. It also discusses the limitations of previous studies. Section 3 describes the proposed framework, including traffic state representation, policy learning, agent coordination, and hierarchical emergency control. Section 4 gives the experimental settings and reports the comparative, ablation, convergence, and emergency-scenario tests. Section 5 discusses the results, practical limitations, and possible extensions. Section 6 concludes the paper and summarizes the main findings.

## 2. Previous studies

### 2.1. Core principle of contrastive predictive coding

Contrastive Predictive Coding (CPC) learns representations of sequential data by predicting future elements rather than reconstructing the input itself. The model uses three parts: a feature encoder, an autoregressive context module, and a prediction function. The encoder first maps the raw observations into latent features. These features are then gradually accumulated by the context module, producing a context vector that contains information from previous observations. GRU or LSTM structures are often employed at this stage to preserve temporal dependencies across multiple steps [9,10]. Based on the resulting context, the prediction function forecasts future latent features. The InfoNCE objective then compares each prediction with its corresponding future feature and with negative samples from other observations [11,12]. This setup is relevant to traffic sequences because vehicle trajectories and intersection flows change over time and are difficult to characterize from isolated measurements alone.

CPC determines how positive and negative pairs are formed during training. A predicted representation and the feature observed at the matching future step form a positive pair, and features taken from other observations are used as negatives. The same approach has been applied in several fields. Speech models predict later frames based on the current context, and image applications compare a predicted local region with representations at other spatial locations [13]. The number of negative samples and prediction horizons can be adjusted according to the complexity of different scenarios, allowing CPC to capture task-relevant information and maintain adaptability across diverse dynamic environments [14]. In traffic applications, such adjustments allow the representation process to account for differences in sequence length, traffic variability, and emergency-event dynamics.

Vehicle movement, traffic congestion, and intersection conditions are continuously changing over time. CPC can represent these observations by accumulating earlier features in its autoregressive context module to obtain context vectors that retain temporal relationships among successive traffic states [15]. This differs from manually designed feature extraction, where the model relies on predefined indicators and rules. Learned CPC representations can instead encode patterns that arise from the observed sequence itself. During an emergency, traffic conditions may change suddenly, and the controller may encounter situations that were not present in the training data. Under these conditions, the compact representation generated by CPC can be used as state input for traffic-control policies that require timely and adaptive estimates of the current traffic condition [16].

### 2.2. Multi-agent meta-reinforcement learning

Multi-agent reinforcement learning combined with meta-learning (MARL+MetaRL) is used for policy adaptation in cooperative traffic control. Several agents can coordinate decisions at intersections, and meta-learning is used to update the policy according to changes in operating conditions [17–19]. Distributed MARL has therefore been examined in large-scale signal control, where agents exchange traffic information and adjust policies jointly across the network [20,21]. Such methods work well when traffic patterns are adequately represented during training, but their behavior is less predictable when interaction data are limited or the encountered situation differs from the training cases. This issue is particularly relevant to emergencies, because incident type, affected area, and congestion propagation can change within a short period. A controller that performs well under routine demand may therefore need to adapt from only a small amount of new information when an unfamiliar emergency occurs.

In current MARL+MetaRL traffic-control designs, an intersection is usually treated as an agent with access to local observations and information received from neighboring controllers. Meta-training exposes these agents to several traffic tasks so that part of the learned policy can be reused, after which fine-tuning modifies the policy for a newly encountered case [22,23]. This procedure reduces the need to train a controller from the beginning whenever traffic conditions change. Its value for emergency control lies in shortening the adjustment process, although the quality of the resulting policy still depends on what the agent observes and how well the training tasks represent the new situation.

Action and reward design have also been studied in emergency-oriented research. Typical operations include changing the signal phase, adjusting green time, and issuing route-guidance instructions. Reward functions generally combine emergency vehicle travel efficiency, delay to regular traffic, and safety constraints [24,25]. These choices help agents organize their behavior but do not reduce the need for relevant state information. If the emergency type, traffic demand, or propagation pattern is significantly different from those used in training, policy adaptation may still be hindered. Compact state descriptions and efficient adaptation are therefore closely related; reducing redundant input is only beneficial if the representation still contains the information needed for control [26,27].

Other studies have also discussed the limitations of MARL+MetaRL in emergency traffic control [28]. Coordination among intersections can prevent agents from making completely independent decisions. Meta-learning also allows earlier experience to be used when traffic conditions change. Many existing implementations still depend on manually defined traffic features. Some of these variables may contain redundant information or contribute little to the control task, which increases the policy input and makes optimization more difficult. Communication also becomes a problem when the number of agents increases. More information must be exchanged between intersections, and this can create considerable overhead in large traffic networks [29]. Emergency-oriented MARL systems therefore need more than rapid policy adaptation. The state representation should remain compact, and coordination should provide useful information without creating excessive communication or optimization costs.

### 2.3. Key requirements for emergency traffic control

Emergency traffic control is sensitive to response delay. A slow change in signal timing can increase the time needed for an emergency vehicle to pass through an intersection. Previous studies have associated delayed response with longer travel times to incident locations and lower rescue effectiveness [30]. Fixed-time control has obvious limitations once an unexpected event occurs after the timing plan has been set. Semi-adaptive schemes can respond to changing conditions, but they still require time to revise the signal plan after an emergency is detected. The difficulty becomes greater when an emergency vehicle travels through several intersections and traffic conditions vary along the route. Traffic information needs to be translated into signal actions within a short period. Neighboring intersections also need to coordinate their decisions to avoid secondary congestion caused by emergency priority [31].

Emergency events also differ greatly from one another. Ambulance passage, traffic accidents, and fire evacuation vary in severity, affected area, and their influence on surrounding traffic. An event may involve only one intersection or spread across several connected intersections. Previous statistics show that about 40% of emergency events extend beyond their initial area, especially when accident-related congestion reaches adjacent intersections [32]. A fixed route or timing plan may no longer be suitable as the situation changes. Control decisions need to follow changes in emergency vehicle trajectories, queue development, and intersection conditions. Trajectory-based adaptive control has been reported to increase the success rate from 60% to 90%, a 30% improvement over strategies based on predefined routes or fixed plans. These results support updating the control policy during the course of an emergency rather than relying only on decisions made at its beginning [33].

Recent studies have used richer traffic information to support policy adaptation. Multi-agent learning can combine several types of traffic observations instead of relying only on a small set of local indicators. Multimodal traffic representations and adaptive decision methods have also been applied to emergency traffic control, and some studies reported better response performance after additional traffic information was introduced [34,35]. A more detailed state description usually requires greater processing effort. Policy adjustment can also become difficult when a new emergency differs from the situations represented in the training data. Lightweight representation learning and meta-adaptive control have received increasing attention for this reason. Their main purpose is to preserve useful traffic information while keeping the computational cost of rapid response within a practical range.

Emergency priority also has to be considered together with its effect on ordinary traffic. Previous studies suggest that the additional delay introduced by emergency intervention should generally remain within about 5% of normal traffic conditions; once the increase approaches or exceeds 8%, congestion can spread to neighboring intersections and degrade network operation [36]. One emergency-control experiment addressed this conflict with different delay thresholds for different priority levels. The permitted increase was no more than 10% for high-priority events and 5% for medium- and low-priority events. Relative to a unified strategy, this hierarchical arrangement reduced traffic interference by 25% while preserving emergency vehicle passage. The comparison shows why event severity cannot be treated as a descriptive label alone. It needs to affect the control policy itself so that emergency response and regular traffic delay are managed under different priority conditions [37].

Traffic signal control methods differ in how they represent traffic states, adapt policies, and coordinate decisions among intersections. Table 1 compares several representative methods with the proposed framework in terms of research focus, traffic state representation, control strategy, emergency scenario treatment, adaptation capability, and current limitations.

**Table 1:** Structured comparison of prior studies and the current study.

Study	Research focus	Traffic state representation	Control method	Emergency scenario consideration	Rapid adaptation capability	Identified limitation
Li et al. [4]	Network-wide traffic signal optimization	Manually defined traffic features	Multi-agent deep reinforcement learning	Not specifically considered	Limited	Dynamic emergency adaptation was not addressed
Devailly et al. [6]	Large-scale traffic signal control	Graph-based traffic states	Inductive graph reinforcement learning	Not specifically considered	Limited	Rapid policy adaptation to unseen emergencies was not examined
Wu et al. [10]	Distributed traffic signal control	Local traffic observations	Distributed deep reinforcement learning	Not specifically considered	Limited	The method requires sufficient training for new traffic conditions
Zhu et al. [11]	Intelligent traffic light coordination	Broad state features	Multi-agent broad reinforcement learning	Not specifically considered	Limited	Emergency priority and rapid adaptation were not jointly considered
Liu et al. [12]	Cooperative control of multiple intersections	Traffic flow and intersection states	Cooperative multi-agent reinforcement learning	Not specifically considered	Limited	The method mainly focuses on routine traffic coordination
Bouktif et al. [13]	Traffic signal control with consistent state and reward design	Predefined state variables	Deep reinforcement learning	Not specifically considered	Limited	Multi-agent emergency coordination was not included
Zeinaly et al. [17]	Resilient intersection control under accident scenarios	Intersection traffic states	Deep reinforcement learning	Accident scenarios	Limited	Fast adaptation to different emergency types was not investigated
Jia et al. [7]	Large-scale traffic signal coordination	Spatiotemporal traffic features	Attention-based multi-agent reinforcement learning	Partially considered	Limited	Meta-learning-based rapid adaptation was not included
Yang et al. [8]	Emergency-responsive traffic signal control	Graph-based traffic information	Multi-agent reinforcement learning	Explicitly considered	Moderate	Generalization to previously unseen emergencies remains limited
Our study	Rapid adaptive traffic control after emergencies	CPC-based low-dimensional contextual representation	Multi-agent meta-reinforcement learning with hierarchical control	Explicitly considered	Strong	Requires further investigation of computational efficiency and real-world deployment under large-scale and diverse emergency scenarios

Table 1 shows that existing approaches have primarily investigated traffic signal optimization, coordination among multiple intersections, or control strategies designed for specific emergency situations. However, many of these methods still depend on manually constructed or conventional traffic state representations and often require additional retraining when traffic conditions vary. The combination of compact traffic representation, rapid adaptation to unfamiliar emergency scenarios, multi-agent coordination, and priority-aware control has received limited investigation in existing frameworks. In this study, Contrastive Predictive Coding is incorporated with multi-agent meta-reinforcement learning and hierarchical emergency control to establish a unified framework for adaptive emergency traffic management.

## 3. Algorithm model design

### 3.1. CPC efficient representation model for traffic states

**Fig 2:**
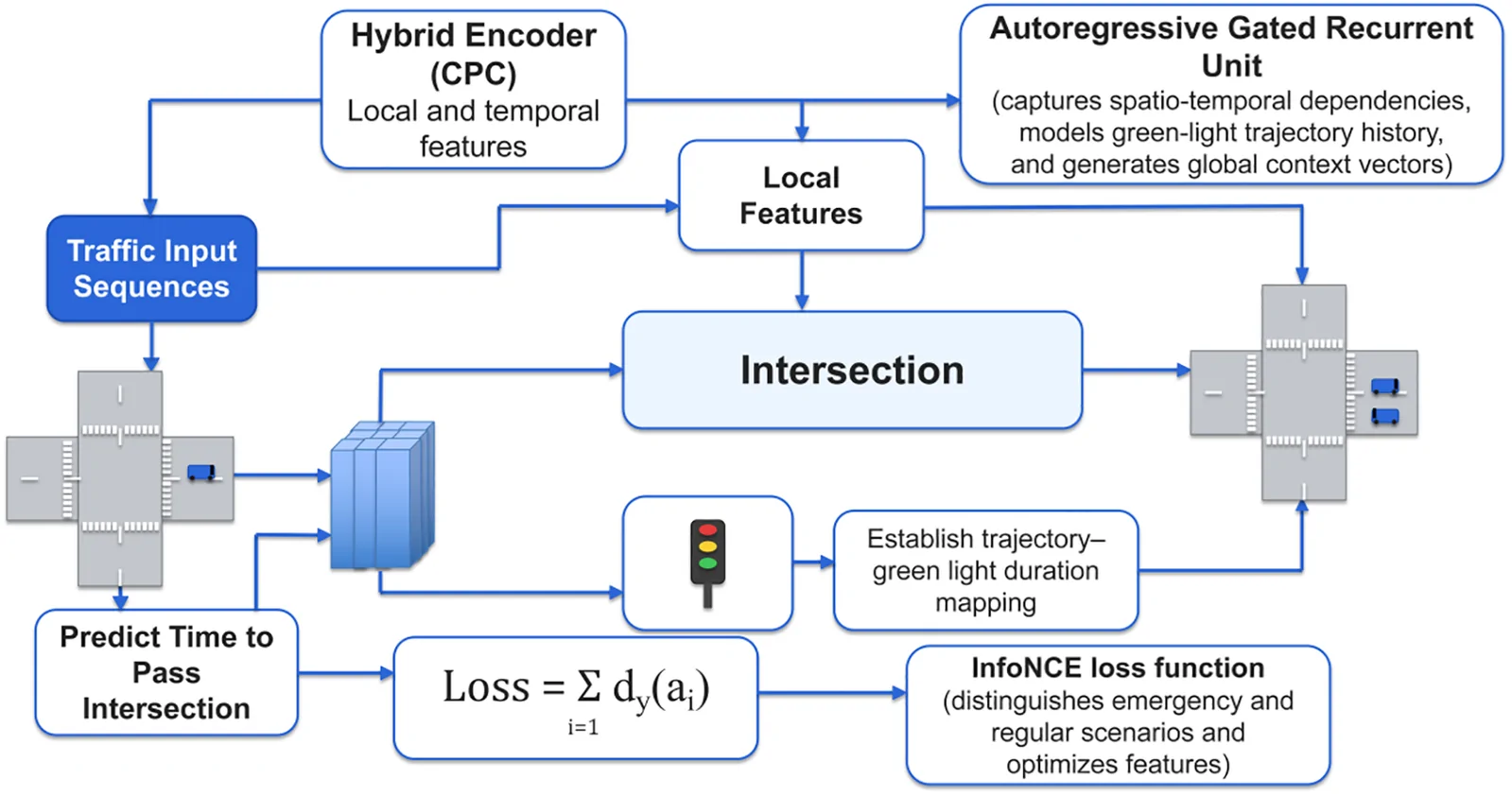
CPC efficient representation model.

The traffic input sequence at each time step is first processed by a hybrid encoder equipped with emergency vehicle identifiers. Convolutional layers are applied to extract spatial location characteristics of emergency vehicles and obtain local feature representations, as illustrated in Fig 2. An autoregressive gated recurrent unit is then used to aggregate temporal dependencies from historical local features, where the forget gate selectively preserves trajectory-related information of emergency vehicles and produces context vectors containing spatiotemporal information. Based on these context vectors and a linear projection matrix, the prediction horizon is defined according to the estimated time required for an emergency vehicle to pass the next intersection. Future local features are predicted to establish the relationship between emergency vehicle trajectories and green-light duration adjustments. The InfoNCE loss is subsequently constructed by computing cosine similarity between real local features and negative samples collected from non-emergency scenarios, encouraging the model to distinguish emergency and regular traffic patterns during representation learning. Through the combination of local feature extraction, temporal context encoding, future feature prediction, and contrastive optimization, the model obtains traffic state representations for subsequent decision-making.

(1) Local feature encoding of traffic state

$$\begin{array}{c} z_{t}=Encoder\left(X_{t}\right) \end{array}$$ (1)

The traffic input sequence at time t;Encoder is the hybrid encoder adapted to the traffic scene; and $z_{t}$ is the local feature vector of traffic at time t. Scene adaptation: For emergencies, $X_{t}$ a binary identifier bit for emergency vehicles is embedded. The encoder captures the spatial location features of emergency vehicles through the convolutional layer to ensure$z_{t}$ rapid identification of emergency scenes [38].

(2) Self-regressive context vector generation

$$\begin{array}{c} c_{t}=AR-GRU\left(z_{t},c_{t-1}\right) \end{array}$$ (2)

In the formula, AR−GRU represents an autoregressive gated recurrent unit, which is used to accumulate the spatiotemporal dependencies of historical local features; $c_{t-1}$ denotes the context vector at time t−1; and $c_{t}$ stands for the global context vector at time t. Scene adaptation: Considering the temporal continuity of traffic flow, AR−GRU the forget gate preferentially retains the historical information of emergency vehicle trajectories, ensuring $c_{t}$ accurate prediction of the future path of emergency vehicles.

(3) Prediction of future transportation characteristics

$$\begin{array}{c} {\hat{z}}_{t+k}=W_{k}\cdot c_{t} \end{array}$$ (3)

When the prediction step length is k; $W_{k}$ is the linear weight matrix for k step prediction; and ${\hat{z}}_{t+k}$ is the local feature vector at the prediction time t+k. Scene adaptation: For emergencies, k set it as the estimated time for emergency vehicles to pass through the next intersection, and $W_{k}$ is learn the mapping relationship of “emergency vehicle trajectory → green light duration adjustment” through training, ensuring that the prediction features can directly support subsequent decision-making [39].

(4) InfoNCE loss adapted to traffic scenarios

$$\begin{array}{c} L_{InfoNCE}=-E\left[log\frac{exp\left({\hat{z}}_{t+k}^{T}z_{t+k}\right)}{exp\left({\hat{z}}_{t+k}^{T}z_{t+k}\right)+\sum_{j=1}^{M}exp\left({\hat{z}}_{t+k}^{T}z_{j}^{-}\right)}\right] \end{array}$$ (4)

In the formula, E[·] denotes the expectation over the sampled traffic sequences and corresponding positive-negative sample pairs during the training process. $z_{t+k}$ represents the true local feature vector at time t+k; $z_{j}^{-}$ represents the feature vector of the j -th negative sample;${\hat{z}}_{t+k}^{T}z_{t+k}$ represents the cosine similarity between the predicted feature and the true feature; and $\sum_{j=1}^{M}exp\left(\cdot \right)$ represents the sum of negative sample similarities. Scene adaptation: Negative samples are preferentially selected from traffic features in non-emergency scenarios, allowing the model to capture discriminative characteristics between emergency and regular traffic conditions and improve emergency recognition accuracy [40].

### 3.2. Multi-agent meta-reinforcement learning decision framework

**Fig 3:**
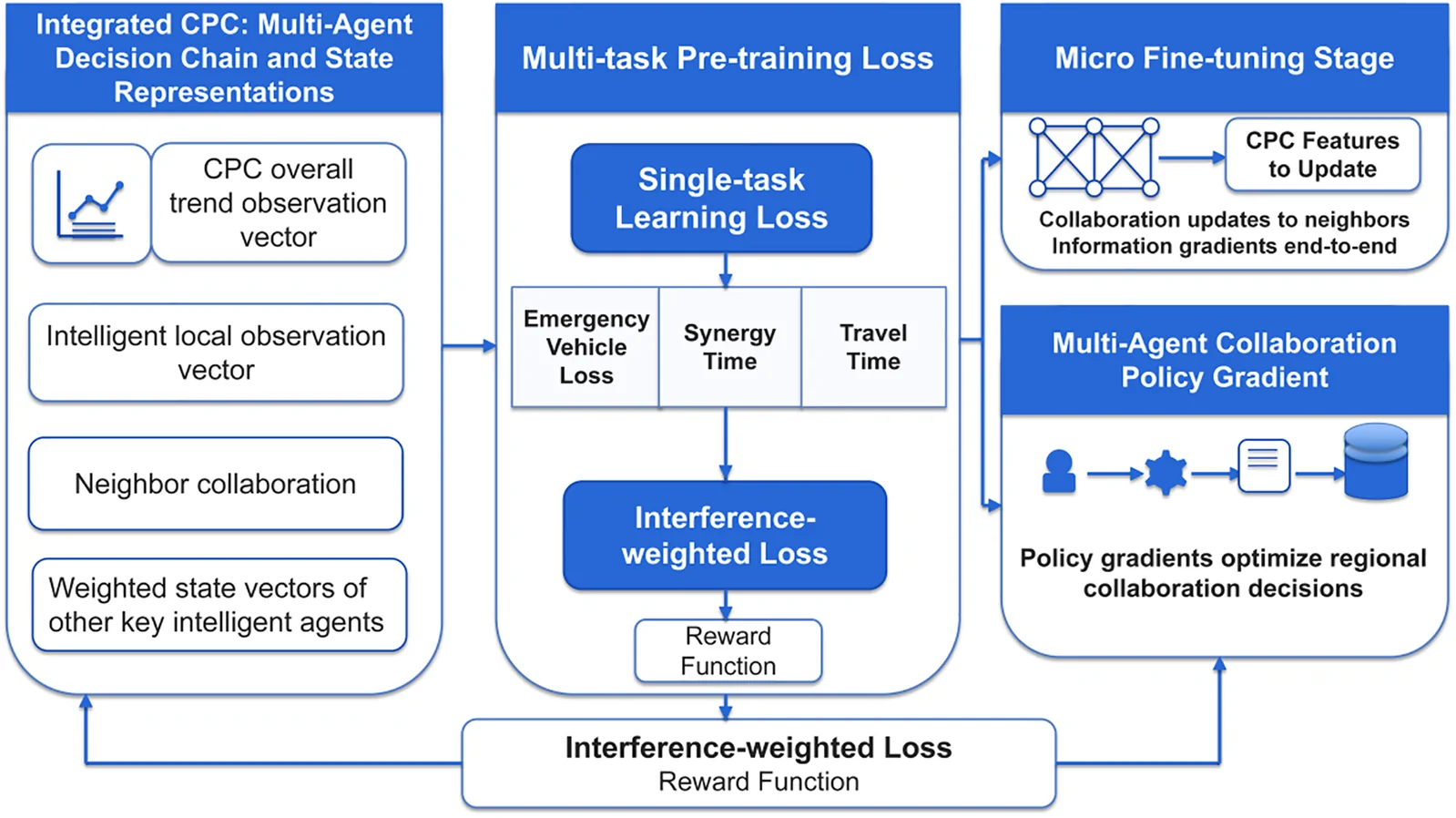
Multi agent meta reinforcement learning.

The multi-agent decision process starts from the state representation integrated with CPC, where the global context vector generated by CPC is combined with agent-level observations and neighboring agent interaction information, as shown in Fig 3. The agent state vector is obtained through weighted aggregation of neighboring states, providing the input representation for subsequent policy optimization. The meta-pretraining stage constructs a multi-task objective based on individual reinforcement learning losses, where each task adopts a reward function that jointly considers emergency vehicle travel time, regular traffic delay, and interference level. The reward formulation is intended to keep emergency response gains from being achieved at the expense of routine traffic operation. During meta-fine-tuning, policy updates combine gradients from neighboring agents with end-to-end refinement of the CPC representation, so that an agent can revise its control policy when it encounters a new emergency case. Cross-agent action information is also included in the collaborative gradient, which links local policy changes to regional coordination and helps avoid isolated convergence toward a local optimum. The resulting update sequence connects state representation, objective optimization, policy adaptation, and collaborative control.

$s_{i}^{t}$ is the state vector of the i -th agent at time step t; $c^{t}$ is the global traffic context vector output by the CPC model; $o_{i}^{t}$ is the local observation vector of the agent; N(i) is the neighbor set of the agent i; $w_{ij}^{t}$ is the communication weight between i -th and j -th agents at time step t; ⊕ is the feature concatenation operation; and $\sum_{j\in N\left(i\right)}w_{ij}^{t}\cdot s_{j}^{t}$ is the weighted aggregation of the states of neighboring agents [41].


$$\begin{array}{c} s_{i}^{t}=\left[c^{t}⊕o_{i}^{t}⊕\sum_{j\in N\left(i\right)}w_{ij}^{t}\cdot s_{j}^{t}\right] \end{array}$$ (5)

Scenario adaptation: By integrating CPC global features with local observations and neighboring collaborative information, the agent can capture global emergency scenarios while precisely controlling local intersections, addressing the “local optimum” problem.

Meta-pretraining objective


$$\begin{array}{c} L_{meta}=E_{\tau \;T}\left[E_{\left(s,a,r,s^{\prime }\right)\;\tau }\left[L_{RL}\left(\theta ;\tau \right)\right]\right] \end{array}$$ (6)

Among them, the loss of single-task reinforcement learning $L_{RL}\left(\theta ;\tau \right)$ is:


$$\begin{array}{c} L_{RL}\left(\theta ;\tau \right)=-E_{s\;p_{\theta },a\;\pi_{\theta }}\left[log\pi_{\theta }\left(a|s\right)\cdot R\left(s,a\right)+\beta \cdot KL\left(\pi_{\theta }\left(\cdot |s\right)\|\pi_{\theta_{old}}\left(\cdot |s\right)\right)\right] \end{array}$$ (7)

Traffic scenario reward function R(s,a):


$$\begin{array}{c} R\left(s,a\right)=\lambda_{\text{1}}\cdot \frac{\text{1}}{T_{emerg}}+\lambda {\;}_{\text{2}}\;\cdot \left(-D_{reg}\right)+\lambda_{\text{3}}\cdot \left(-P_{disturb}\right) \end{array}$$ (8)

T denotes the set of all atomic tasks; τ is a single atomic task; θ is the atomic policy parameter; $p_{\theta }$ denotes the state visitation distribution induced by policy $\pi_{\theta }$;$\pi_{\theta }\left(a|s\right)$ is the agent policy; $T_{emerg}$ is the time for emergency vehicles to pass through the current area;$D_{reg}$ is the delay for regular vehicles; $P_{disturb}$ is the degree of interference of emergency control on regular traffic; $\lambda_{\text{1}}/\lambda {\;}_{\text{2}}\;/\lambda_{\text{3}}$ are weighting coefficients for emergency vehicle efficiency, regular vehicle delay, and traffic interference, respectively; and β is the KL divergence coefficient [42].

Scenario adaptation: Meta-pretraining obtains transferable policies from diverse emergency tasks, allowing agents to adjust their strategies when encountering new emergency scenarios with only a limited number of samples. This mechanism reduces the dependence on extensive interaction data and improves the real-time adaptation capability of emergency traffic control.

Collaborative strategy update in the meta-tuning phase


$$\begin{array}{c} \theta_{i}^{\tau }=\theta_{i}-\alpha_{\text{1}}\cdot \nabla_{\theta_{i}}L_{RL}\left(\theta_{i};\tau \right)+\alpha {\;}_{\text{2}}\;\cdot \nabla_{\theta_{i}}\sum_{j\in N\left(i\right)}L_{RL}\left(\theta_{i};\tau \right) \end{array}$$ (9)

Among them, the gradient term$\nabla_{\theta_{i}}L_{RL}\left(\theta_{i};\tau \right)$ includes end-to-end update of CPC features:


$$\begin{array}{c} \nabla_{\theta_{i}}L_{RL}\left(\theta_{i};\tau \right)=\nabla_{\theta_{i}}\left[-log\pi_{\theta }\left(a|s{\;}_{i}{\;}^{t}\right)\cdot R\left(s{\;}_{i}{\;}^{t},a\right)\right]+\gamma \cdot \nabla_{c^{t}}s{\;}_{i}{\;}^{t}\cdot \nabla_{\theta_{CPC}}c^{t} \end{array}$$ (10)

In the formula, $\theta_{i}^{\tau }$ represents the updated policy parameter of agent i after adaptation to current emergency task τ;$\alpha_{\text{1}}/\alpha {\;}_{\text{2}}\;$ denotes the fine-tuning learning rate/co-learning rate; N(i) denotes the set of neighboring agents connected with agent i; $\nabla_{\theta_{i}}\sum_{j\in N\left(i\right)}L_{RL}\left(\theta_{i};\tau \right)$ signifies the loss gradient of the neighboring agents; $\theta_{CPC}$ denotes the trainable parameters of the CPC encoder; $\nabla_{c^{t}}s{\;}_{i}{\;}^{t}$ represents the gradient of the agent state with respect to the global context vector $c^{t}$; and γ is the CPC parameter update coefficient [43].

Scenario adaptation: Collaborative fine-tuning and end-to-end updating of CPC parameters allow agents to adjust their policies under new emergency scenarios while simultaneously improving the consistency between learned CPC representations and subsequent decision-making processes.

Multi-agent collaborative strategy gradient


$$\begin{array}{c} \nabla_{\theta }J\left(\theta \right)=E_{s\;p_{\theta },a\;\pi_{\theta }}\left[\sum_{i=1}^{K}\nabla_{\theta_{i}}log\pi_{\theta_{i}}\left(a_{i}|s_{i}\right)\cdot \left(Q_{i}\left(s,a\right)-V_{i}\left(s_{i}\right)+\sum_{j\neq i}\nabla_{a_{j}}Q_{i}\left(s,a\right)\cdot \nabla_{\theta_{i}}\pi_{\theta_{j}}\left(a_{j}|s_{j}\right)\right)\right] \end{array}$$ (11)

K is the number of agents;$a=\left(a_{\text{1}},\text{\textbackslash{}ldots},a_{K}\right)$ is the joint action of multi-agent;$s=\left(s_{\text{1}},\text{\textbackslash{}ldots},s_{K}\right)$ is the joint state of multi-agent; $Q_{i}\left(s,a\right)$ is the joint action value function of agent; $V_{i}\left(s_{i}\right)$ is the state value function of agent; $\nabla_{a_{j}}Q_{i}\left(s,a\right)\cdot \nabla_{\theta_{i}}\pi_{\theta_{j}}\left(a_{j}|s_{j}\right)$ is the gradient influence of agent i on agent j action; j≠i indicates the influence of other agents except agent i during collaborative policy updating. Scenario adaptation: Traditional multi-agent policy gradients only consider the impact of individual actions. By incorporating the “cross-agent action gradient” into this formula, collaborative control within the region can be optimized [44].

**Fig 4:**
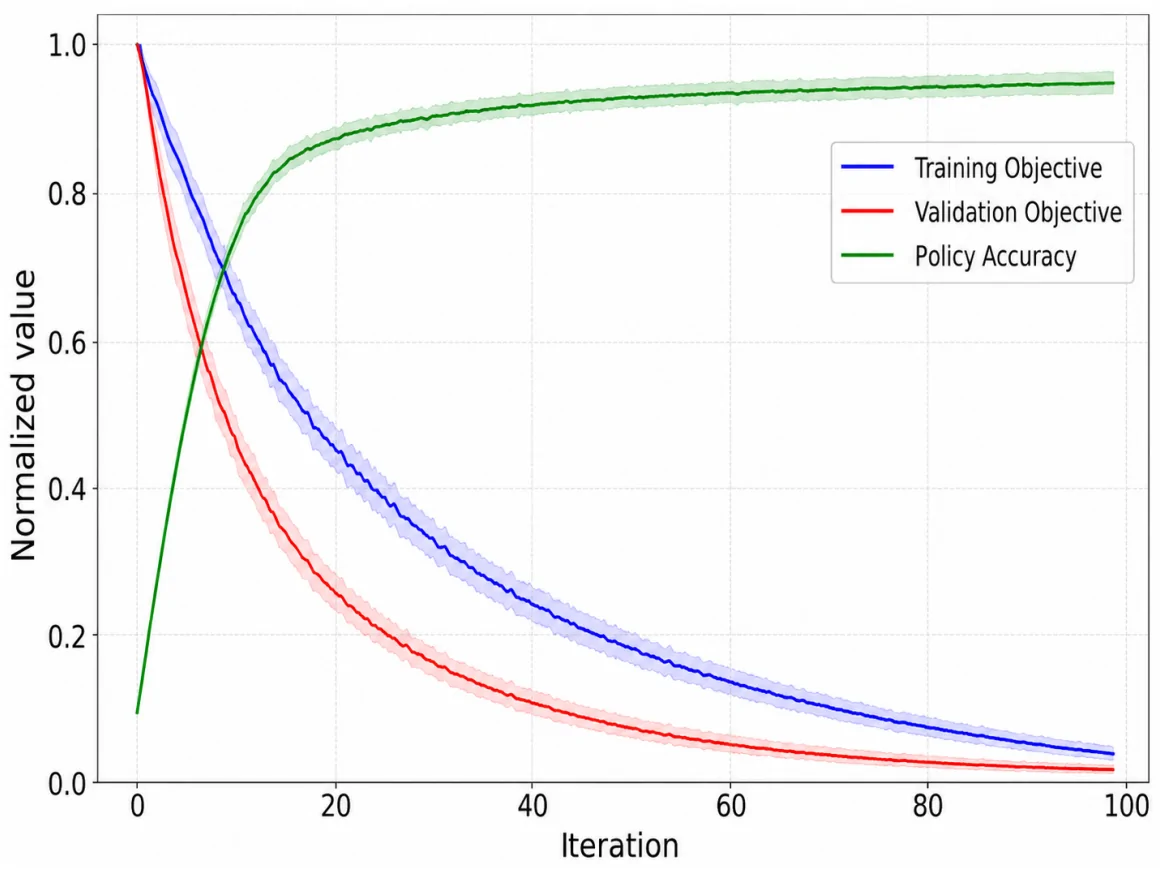
Optimization convergence of the proposed CPC-MetaMARL framework.

Fig 4 presents the convergence curve of the proposed framework during the optimization process. The iterative curve illustrates the variation of the objective value during the training process and provides an indication of optimization behavior and policy convergence. As the number of iterations increases, the objective value gradually decreases and becomes stable within a certain range, indicating that the learning process reaches a relatively steady state under emergency traffic scenarios. The convergence trend also reflects the interaction between CPC-based representation learning and multi-agent meta-reinforcement learning during policy optimization. No evident oscillations are observed in the later training stages, suggesting that the proposed framework maintains stable parameter updates throughout the optimization process.

### 3.3. Hierarchical emergency priority control

After meta-adaptation, the learned policy is passed to a priority-aware control layer. Each detected emergency is assigned a high, medium, or low priority according to the scenario type and event severity. The priority level does not replace the learned policy; instead, it adjusts the control intensity used when selecting signal actions. High-priority cases receive the strongest passage intervention, whereas medium- and low-priority cases use progressively milder adjustments to limit unnecessary disturbance to regular traffic. This priority-dependent mechanism is the same control logic evaluated in the hierarchical-control ablation and the cross-scenario experiments [45].

The CPC-MetaMARL algorithm integrates traffic representation learning with emergency-oriented adaptive control through a sequential optimization process. The main procedure is summarized in Table 2. The algorithm first initializes the CPC encoder, prediction module, and multi-agent policy parameters, which provide the basis for subsequent traffic feature extraction and control policy optimization. The CPC component obtains compact traffic representations through local feature encoding, temporal context generation, future representation prediction, and InfoNCE loss optimization. The resulting CPC-enhanced states are then provided to the multi-agent meta-reinforcement learning framework, where multiple emergency tasks are used during meta-training to learn transferable control strategies. When encountering unfamiliar emergency scenarios, agents perform few-shot adaptation through policy updates together with CPC representation refinement. Afterward, collaborative policy optimization and hierarchical emergency priority control determine the traffic signal actions for different emergency conditions. This algorithm establishes an end-to-end learning procedure that reduces redundant traffic information, improves adaptation under emergency scenarios, and maintains a balance between emergency vehicle priority and regular traffic disruption.

**Table 2:** Proposed CPC-MetaMARL algorithm.

Input:	Traffic sequence X; emergency task set T; number of agents N; learning rates A1 and A2; CPC update rate G; prediction horizon set K; negative sample count M.
**Output**:	Optimized emergency control policy P and CPC parameter set C.
1	Initialize CPC encoder parameter set C, prediction matrices Wk, and agent policy parameters Pi for all agents i from 1 to N.
2	For each traffic sequence X at time t, encode local representation Zt by applying the CPC encoder with parameter set C.
3	Generate temporal context Ct from Zt and the previous context using the autoregressive GRU.
4	For each prediction horizon k in set K, predict future representation Zp from prediction matrix Wk and context Ct.
5	Update parameter set C and prediction matrices Wk by minimizing InfoNCE objective Lc over positive and negative sample pairs.
6	For each emergency task q in set T and agent i, construct state Si from CPC context Ct, local observation Oi, and the distance and delay weighted neighboring state aggregate based on Wij.
7	Compute meta training loss Lm by aggregating task losses Lq across all tasks in set T, and update the shared meta policy parameters.
8	Adapt each agent to the current task by updating policy parameter Pi with learning rate A1 and the task loss gradient.
9	Refine CPC encoder parameter set C end to end using update rate G and the CPC loss gradient.
10	Aggregate collaborative gradients from neighboring agents with co learning rate A2 and update the joint policy using multi agent objective J.
11	Assign emergency priority R as high, medium, or low, and execute the corresponding signal control action Ai for agent i.
12	Return adapted agent policies Pi for all agents together with the optimized CPC parameter set C.

## 4. Research experimental results

### 4.1. Experimental platform and dataset

The experiments are conducted on the iTETRIS v1.1 simulation platform, which combines SUMO v1.15.0 for traffic flow modeling and ns-3 v3.35 for communication simulation to establish a traffic-communication co-simulation environment. In the SUMO environment, the traffic scenario is constructed using a downtown road network with a latitude and longitude range of 113.92°E–113.95°E and 22.54°N–22.57°N. The scenario contains 12 signal-controlled intersections, including four four-way intersections and eight three-way intersections. The road lengths vary from 200 to 500 m, and each entrance contains 2–3 lanes. Vehicle generation follows a Poisson distribution, with densities of 180 vehicles/km during peak periods (7:00–9:00 and 17:00–19:00) and 120 vehicles/km during non-peak periods. Emergency vehicles, including ambulances and fire trucks, account for 3% of the total vehicles. Ns-3 supports emulation of V2I and V2V communication according to the IEEE 802.11p standard. Transmission Power is 23 dBm, channel bandwidth is 10 MHz, packet size is 512 bytes, and the communication delay threshold of this setup is 50 ms. Inter-agent information is transmitted in a distance-weighted broadcast mode. Experimental runs are carried out on a workstation with an Intel Xeon Gold 6330 CPU (2.0 GHz, 32 cores), 128 GB of DDR4 memory, and an NVIDIA RTX 3090 GPU (24 GB VRAM), and Ubuntu 20.04 LTS is used as the operating system.

The surveillance videos are 1920 × 1080 in resolution and 25 fps. The annotations include the path of emergency vehicles, vehicle IDs, types, speeds and positions, as well as intersection traffic data and accident information. Entrance-lane queue length samples were taken every 5 seconds, and accident labels recorded the time and location of each occurrence. A total of 500 annotated emergency events were obtained for model training and testing.

Data Preparation had three primary steps. For each intersection entrance lane, a 320 × 240-pixel Region of Interest (ROI) was first extracted, and then the standard deviation of its grey-level histogram was computed as a local traffic descriptor. V2I records containing vehicle ID, emergency status and speed were then combined with ROI-derived visual features to build temporal sequences using a window length of T = 10 seconds. Finally, at 70%, 20% and 10%, the processed data were divided into the training, validation and test sets. These subsets have 70 hours and 350 emergency events, 20 hours and 100 events, and 10 hours and 50 events, respectively. The test set has 15 high-priority, 20 medium-priority and 15 low-priority events.

**Fig 5:**
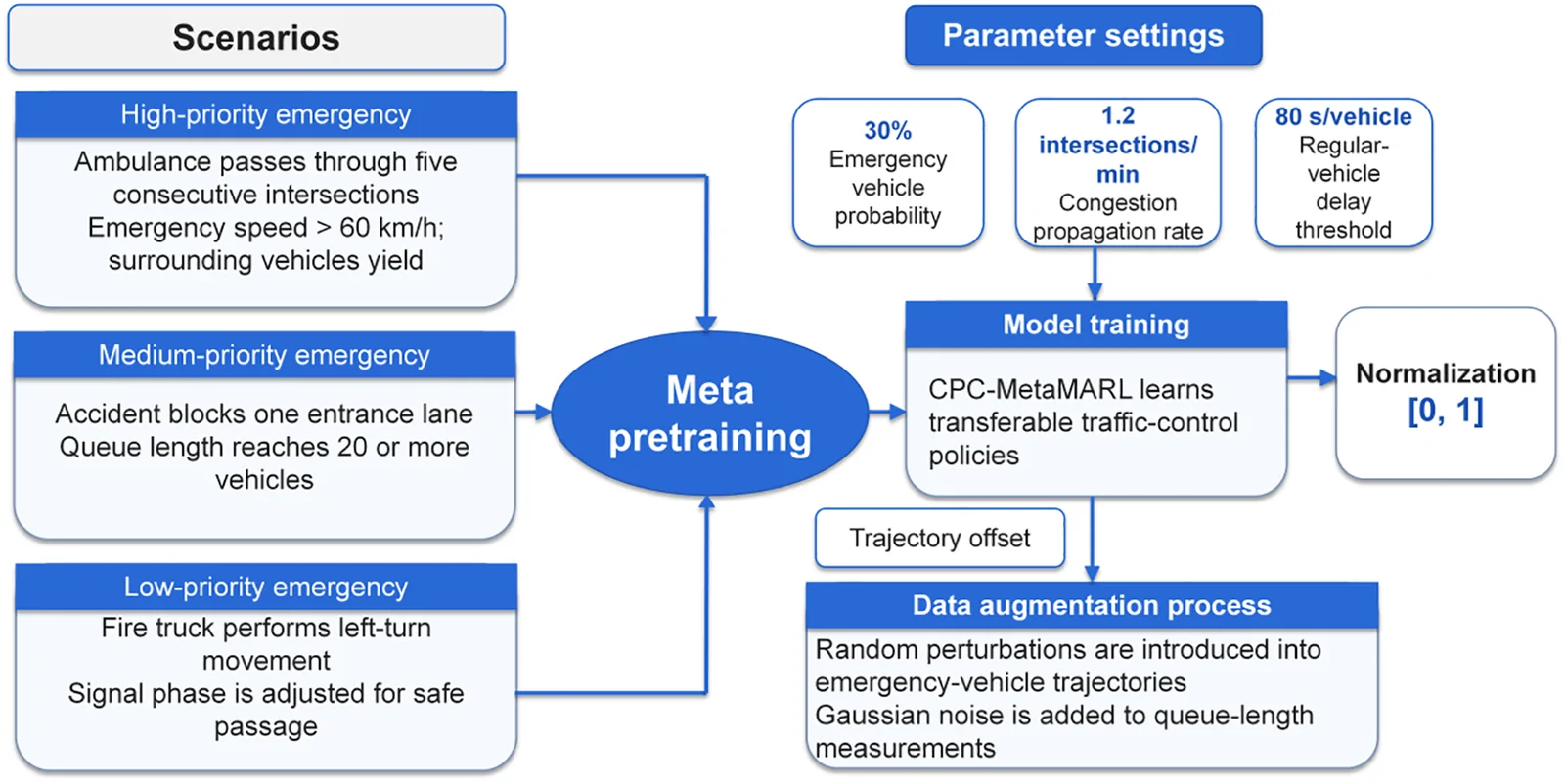
Data collection.

A second set of emergency samples was generated via the SUMO TraCI interface for the meta-pretraining stage. Three event kinds were created. High-priority cases are ambulances passing through five consecutive intersections at a speed exceeding 60 km/h, and surrounding vehicles yield. Medium-priority cases are accidents that block one entrance lane and cause a queue of 20 or more vehicles. Low-priority cases are left-turn movements for fire trucks and the corresponding signal-phase adjustment. There are 1000 samples for each category, and all of them include 5 minutes of traffic data, such as emergency vehicle trajectories and intersection statuses. The simulation uses a 30% emergency vehicle occurrence probability, a congestion propagation rate of 1.2 intersections per minute, and a regular vehicle delay threshold of 80 seconds per vehicle. These synthetic samples are used during meta-pretraining so that the training tasks cover different emergency conditions, as shown in Fig 5.

Before training, feature values in all datasets were scaled to the [0,1] range. Training samples were then augmented by introducing random perturbations into emergency vehicle trajectories and adding Gaussian noise to queue-length measurements. The simulation and dataset parameters followed settings commonly used in traffic engineering studies, with reference to relevant provisions of the Code for Urban Road Traffic Planning and Design. Applying the same preprocessing procedures and parameter rules across experiments keeps different test conditions comparable and makes the reported simulation-based evaluation easier to reproduce under the stated configuration.

### 4.2. Comparative experiment setup

**Fig 6:**
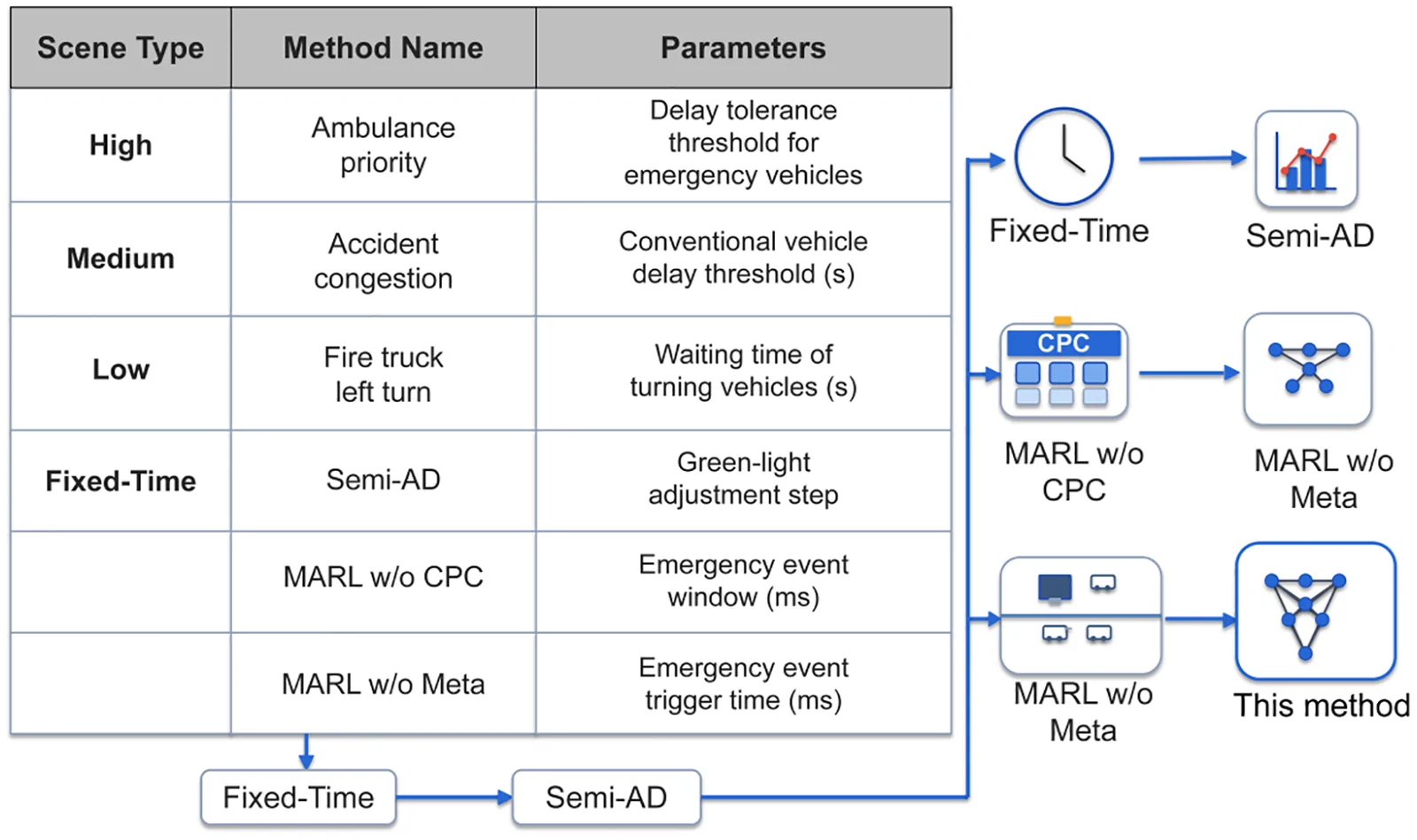
Core control parameters.

Table 3 compares the control settings used by the proposed method and the baseline approaches for emergency traffic scenarios. For high-priority events, the proposed method uses a regular-vehicle delay tolerance of 80 s/vehicle, a 2 s green-light adjustment step, and a 100 ms emergency-event trigger response time. These settings are more responsive than those of the comparison methods and are consistent with the stronger intervention required for high-priority emergencies. Medium- and low-priority events use different parameter values according to their severity, as shown in Fig 6. The controller therefore does not apply one parameter set to every emergency; its operating limits change with event priority to preserve emergency passage while limiting additional delay to regular traffic.

**Table 3:** Core control parameters for various methods under different emergency scenarios.

Scene Type	method name	Delay tolerance threshold for conventional vehicles (s/vehicle)	Green light adjustment step length (s)	Emergency event trigger response time (ms)
High priority (ambulance)	Fixed-Time	None (fixed delay)	0	none
	Semi-AD	120	5	500
	MARL w/o CPC	100	3	200
	MARL w/o Meta	90	3	150
	this method	80	2	100
Medium priority (accident congestion)	Fixed-Time	none	0	none
	Semi-AD	100	5	600
	MARL w/o CPC	90	3	250
	MARL w/o Meta	85	3	200
	this method	75	2	120
Low priority (fire truck turning left)	Fixed-Time	none	0	none
	Semi-AD	80	5	700
	MARL w/o CPC	70	3	300
	MARL w/o Meta	65	3	250
	this method	60	2	150

Table 4 compares the input-output dimensions and state representations of the proposed framework with those of existing approaches. The MARL baseline without CPC uses a 1024-dimensional state vector. This increases the amount of information processed during policy learning and may introduce redundant features. CPC maps traffic states into 256-dimensional latent representations that retain information needed for control decisions. In addition, the action representation is extended to six dimensions, including signal phase selection and coordination operations, and emergency-related features incorporate up to five dimensions of neighboring agent interaction information. The comparison indicates that CPC reduces the dimensionality of traffic state representations and provides a more compact feature space for multi-agent policy optimization, while maintaining the necessary information for emergency control decisions.

**Table 4:** Data table of input and output dimensions and state representations for each method.

Method name	Input feature dimension	State vector dimension	Action space dimension	Emergency feature embedding dimension	Number of output control
Fixed-Time	0 (No input)	0 (manual rule)	0 (fixed instruction)	0	1 (Fixed green light)
Semi-AD	8 (coil detection + queuing)	8 (Artificial features)	2 (extend/do not extend)	1 (Congestion sign)	2
MARL w/o CPC	16 (Artificial feature set)	1024 (high-dimensional artificial)	4 (phase + duration)	2 (Congestion + Emergency Sign)	4
MARL w/o Meta	16 (CPC input)	256 (CPC features)	4 (phase + duration)	3 (Congestion + Emergency + Track)	4
this method	16 (CPC input)	256 (CPC + Collaboration)	6 (Phase + Duration + Linkage)	5 (Congestion + Emergency + Trajectory + Grading + Neighborhood)	6

Table 5 presents the amount and distribution of data used during model training. The complete training set contains 100,000 samples, including 20,000 samples related to emergency events. Meta-pretraining uses data from 10 different scenario types. A separate set of 1,000 samples is used for policy adaptation during the fine-tuning stage. Meta-pretraining allows the model to learn recurring traffic patterns from several emergency conditions. Fine-tuning uses a smaller sample set to adjust the learned policy when a newly encountered emergency case appears. The input data are normalized before training, and data augmentation is applied to increase sample variation. These preprocessing operations reduce differences among individual samples and keep the training and evaluation conditions consistent across the comparative experiments.

**Table 5:** Training data volume and sample distribution for each method.

method name	Total samples	Number of event	Number of tasks	Fine-tune size	Number of (categories)
Fixed-Time	0 (no training)	0	0	0	0
Semi-AD	0 (no training)	0	0	0	0
MARL w/o CPC	50,000	10,000	0	0	5
MARL w/o Meta	50,000	10,000	0	0	5
this method	100,000	20,000	10	1,000	10

Table 6 presents the main simulation settings adopted for the emergency traffic experiments. Each simulation episode lasts 30 minutes and covers emergency occurrence, traffic evolution, and the control response that follows. Emergency vehicle speed is set according to event priority. High-priority cases use 60 km/h, medium-priority cases use 40 km/h, and low-priority cases use 30 km/h. These speed settings represent the different operating conditions included in the experiments. Vehicle-to-intersection communication uses the IEEE 802.11p protocol. The average communication latency is set to 50 ms. Simulation execution and model computation are performed on a platform equipped with a 32-core CPU and a GPU with 24 GB of memory. The normalization procedure described above is used for all experimental data. The same data augmentation procedure is also retained in every experiment. Using the same preprocessing and simulation settings keeps the comparative tests under consistent experimental conditions.

**Table 6:** Detailed values of experimental environment and simulation parameters.

parameter category	parameter name	specific numerical value
Traffic simulation parameters	Simulation duration (for each scenario)	30 minutes (1800 seconds)
	Emergency vehicle speed (high priority)	60km/h
	Emergency vehicle speed (medium priority)	40km/h
	Emergency vehicle speed (low priority)	30km/h
	Average speed of conventional vehicles	25km/h
	Regular vehicle density during peak hours	180 vehicles/km
	Regular vehicle density during off-peak hours	120 vehicles/km
Communication simulation parameters	communication protocol	IEEE802.11p
	Average communication delay	50ms
	Packet loss rate	<2%
	Communication distance threshold among intelligent agents	500m
Experimental hardware parameters	Number of CPU cores	32 cores
	GPU memory	24GB
	simulation step size	1 second

The numerical values reported in the tables are taken from the iTETRIS experimental setup, the CityFlow Emergency dataset, and the parameter settings adopted for the comparison methods. The simulation parameters and reinforcement-learning evaluation procedures use the configurations defined in this study. Traffic-engineering criteria, including the Evaluation Indicator System for Urban Road Traffic Management, are used as references for the experimental settings.

### 4.3. Ablation experiment setup

**Fig 7:**
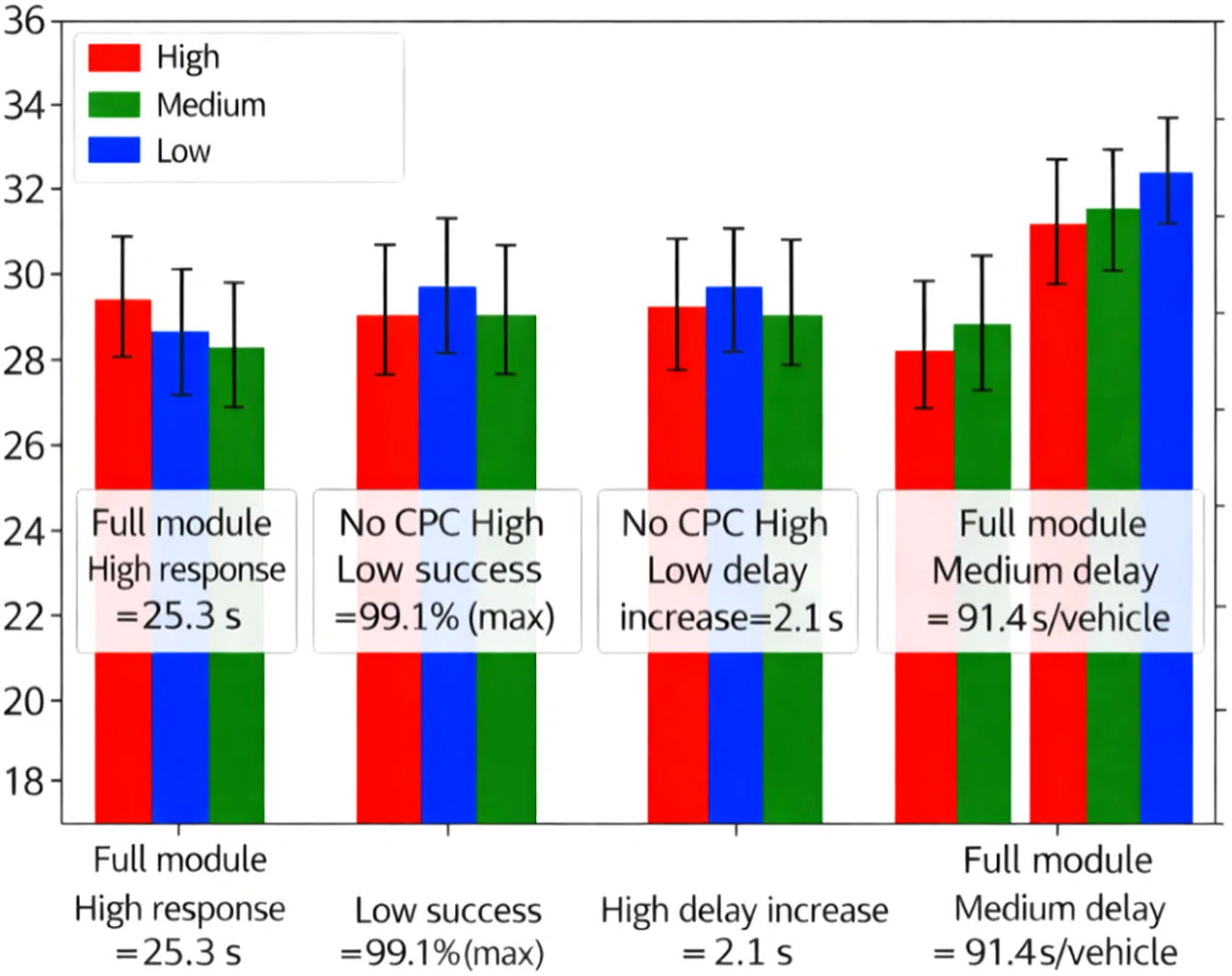
Performance comparison.

Table 7 reports the ablation results for the main components of the proposed framework. The complete model records a high-priority emergency response time of 25.3 s, an average regular-vehicle delay of 80.5 s per vehicle, and an emergency success rate of 98.2%. Removing CPC produces the largest change in response time. The value increases to 35.7 s, which is 41.1% higher than that of the complete model. This result is related to the use of CPC for providing compact traffic-state information during policy optimization. Removing the Meta module mainly affects adaptation performance. The emergency success rate decreases to 92.4% under unfamiliar emergency conditions. Removal of the collaborative module produces a 13.5% increase in regular-vehicle delay. The increase reflects weaker coordination among neighboring agents. Excluding hierarchical control raises the regular-traffic delay increase rate to 10.1%. Emergency priority is still maintained, but the effect on ordinary traffic becomes greater. Fig 7 presents the performance differences among these ablation settings and allows the contribution of each component to be compared directly.

**Table 7:** Performance comparison after ablation of core modules.

Method variant	Scene Type	Emergency response time (s)	Regular average delay (s/vehicle)	Emergency response success rate (%)	Regular delay increase rate (%)
This method (full module)	High priority	25.3 ± 1.2	80.5 ± 3.1	98.2 ± 0.8	4.8 ± 0.5
No CPC module (manual feature substitution)		35.7 ± 2.1	95.2 ± 4.2	85.6 ± 1.5	12.3 ± 1.1
No Meta module (conventional MARL)		30.1 ± 1.5	88.7 ± 3.5	92.4 ± 1.0	7.6 ± 0.7
No multi-agent collaboration (single agent)		32.9 ± 1.8	91.4 ± 3.8	89.7 ± 1.2	9.8 ± 0.9
No hierarchical control (unified strategy)		28.5 ± 1.4	90.3 ± 3.6	94.1 ± 0.9	10.1 ± 0.8
This method (full module)	medium priority	22.4 ± 1.0	75.3 ± 2.8	97.5 ± 0.7	3.5 ± 0.4
No CPC module		31.2 ± 1.9	87.6 ± 3.3	83.2 ± 1.3	9.7 ± 0.8
No Meta module		26.8 ± 1.3	81.5 ± 3.0	91.8 ± 1.1	5.2 ± 0.6
No collaboration module		29.5 ± 1.6	84.2 ± 3.2	88.9 ± 1.2	7.4 ± 0.7
No grading module		25.1 ± 1.2	82.7 ± 3.1	93.6 ± 0.8	6.8 ± 0.6
This method (full module)	low priority	18.2 ± 0.9	60.1 ± 2.2	99.1 ± 0.5	2.1 ± 0.3
No CPC module		25.6 ± 1.4	72.3 ± 2.7	90.5 ± 1.0	6.5 ± 0.5
No Meta module		21.3 ± 1.1	65.4 ± 2.4	95.7 ± 0.6	3.8 ± 0.4
No collaboration module		23.7 ± 1.3	68.9 ± 2.6	93.2 ± 0.7	5.1 ± 0.4
No grading module		19.8 ± 1.0	63.7 ± 2.3	96.8 ± 0.6	3.2 ± 0.3

Table 8 presents the sensitivity results for several key parameters of the CPC module. Among the tested combinations, k = 5, M = 100, and an InfoNCE weight of 1.0 give the shortest emergency response time of 25.3 s and the highest feature adaptability of 95.6%. Setting k to 3 reduces the ability of the model to capture temporal patterns related to vehicle trajectories. A value of k = 7 increases computational cost but does not produce an obvious performance gain. The number of negative samples produces a similar change. M = 50 provides fewer negative examples and limits the diversity of the contrastive set. M = 150 increases the training burden and reduces computational efficiency. The InfoNCE weight also affects the separation between emergency-related and regular traffic representations. An unsuitable weight weakens this separation. The combination k = 5, M = 100, and weight = 1.0 gives the best overall result among the tested settings and retains representation performance at a reasonable computational cost.

**Table 8:** Ablation table of CPC module parameter sensitivity.

CPC parameter combination (k, M, InfoNCE weight)	Emergency response time (s)	Feature vector dimension	Training time (h)	Adaptability of features to decision-making (%)
k=3, M=50, weight=0.5	28.7 ± 1.3	256	12.5	82.3 ± 1.2
k=5, M=100, Weight=1.0	25.3 ± 1.2	256	15.2	95.6 ± 0.8
k=7, M=150, Weight=1.5	26.1 ± 1.2	256	18.7	93.4 ± 0.9
k=5, M=50, weight=1.0	27.5 ± 1.3	256	13.8	90.1 ± 1.0
k=5, M=150, Weight=1.0	25.8 ± 1.2	256	16.9	94.2 ± 0.8
k=5, M=100, weight=0.5	27.2 ± 1.3	256	14.1	89.7 ± 1.1
k=5, M=100, weight=1.5	26.5 ± 1.2	256	16.3	92.8 ± 0.9

**Fig 8:**
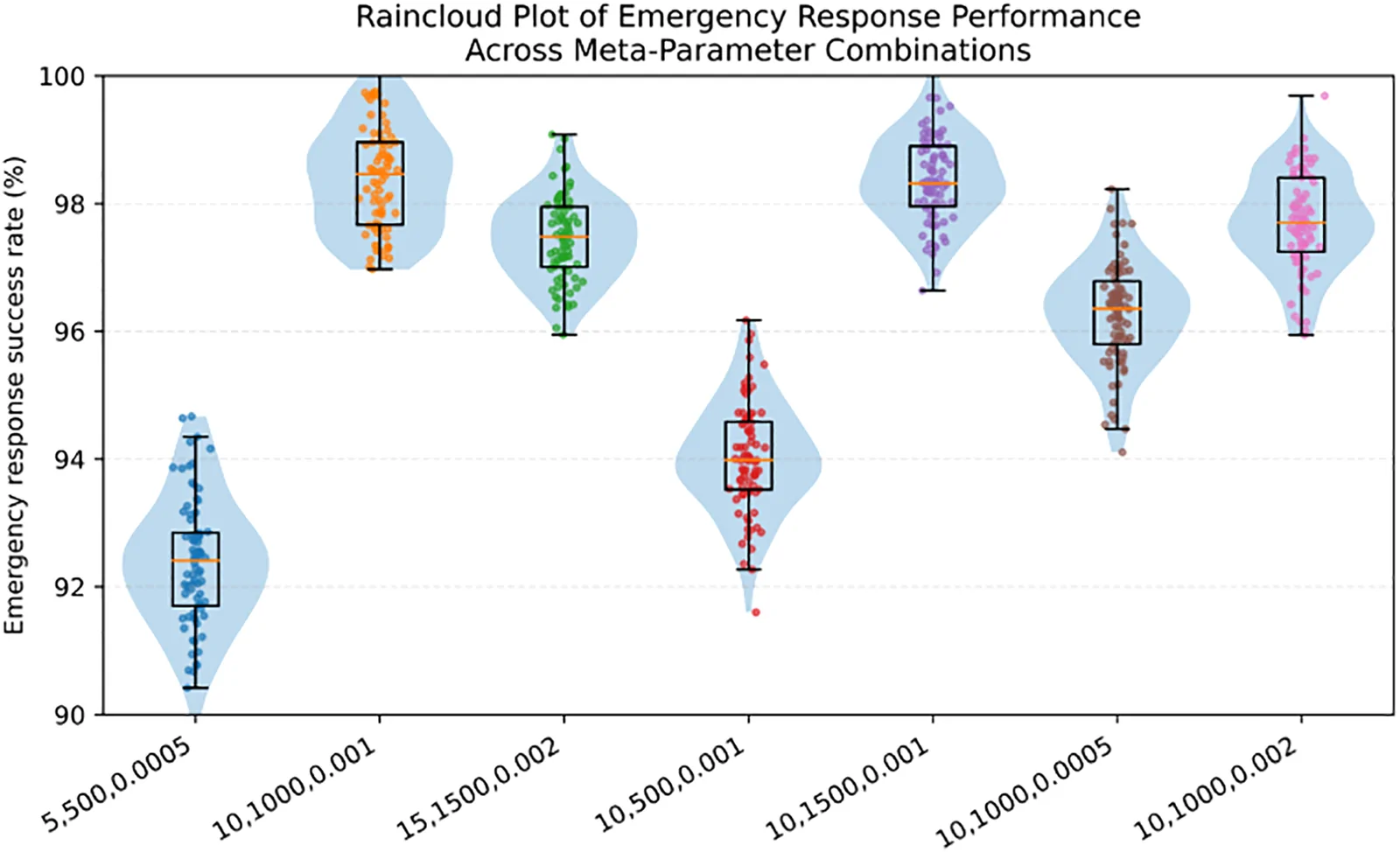
Raincloud Plot of Emergency.

Table 9 reports the sensitivity of the Meta module to several training choices. The combination of 10 pre-training tasks, 1000 fine-tuning samples, and α1 = 0.001 gives the strongest overall adaptation results among the tested settings. With this setup, fine-tuning takes 20.5 min, the emergency success rate reaches 98.2%, the generalization score is 96.8%, and adaptation requires 5.1 s. Using only 5 pre-training tasks exposes the model to fewer emergency patterns and limits the information that can be transferred to a new task. Raising the number to 15 increases training effort but brings no clear additional gain. The number of fine-tuning samples shows a similar trade-off. With 500 samples, the policy receives too little information for reliable adjustment, whereas 1500 samples reduce the practical benefit of few-shot adaptation. Policy updates are also sensitive to the choice of α1, and unsuitable values slow or disturb parameter adjustment. Fig 8 shows how performance changes as these settings vary. Across the tested ranges, the CPC-based multi-agent meta-reinforcement learning framework changes only moderately. The selected configuration therefore represents a practical setting that preserves adaptation performance without introducing unnecessary training cost or marked instability.

**Table 9:** Ablation table of meta module parameter sensitivity.

Meta parameter combination (number of pre-training tasks, number of fine-tuning samples, α_1_)	Fine adjustment time (min)	Emergency response success rate (%)	Cross-scenario generalization score (%)	Adaptation time for new scene (s)
5,500,0.0005	15.2 ± 0.8	92.4 ± 1.0	85.7 ± 1.3	12.3 ± 0.7
10,1000,0.001	20.5 ± 1.1	98.2 ± 0.8	96.8 ± 0.6	5.1 ± 0.3
15,1500,0.002	25.8 ± 1.3	97.5 ± 0.7	95.2 ± 0.8	6.2 ± 0.4
10,500,0.001	17.3 ± 0.9	94.1 ± 0.9	90.3 ± 1.1	7.8 ± 0.5
10,1500,0.001	22.7 ± 1.2	98.5 ± 0.7	97.2 ± 0.6	4.8 ± 0.3
10,1000,0.0005	19.1 ± 1.0	96.3 ± 0.8	93.4 ± 0.9	6.5 ± 0.4
10,1000,0.002	21.3 ± 1.1	97.8 ± 0.7	94.7 ± 0.8	5.5 ± 0.3

The multi-agent collaborative weight ablation experiment verifies the effectiveness of the distance decay + delay weighting mechanism. As shown in Table 10, when σ = 200 (distance threshold) and the delay weighting coefficient = 0.5, the joint reward value reaches 95.6 ± 0.8, the communication delay is controlled at 50.1 ± 2.5ms, the emergency response time is 25.3 ± 1.2s, and the decision consistency is 95.2 ± 0.8%. Too small a σ (100) narrows the collaborative range, leading to local optima, while too large a σ (300) increases communication delay; a lower weighting coefficient (0.2) ignores the conventional delay constraints, and a higher one (0.8) weakens the influence of distance on collaborative priority. This result clarifies the reasonable boundary of collaborative weights and provides a parameter basis for multi-agent linkage.

**Table 10:** Ablation table of multi-agent collaborative weights.

Collaborative weight parameter (σ, delay weighting coefficient)	Joint return value	Communication delay (ms)	Emergency response time (s)	Multi-agent decision consistency (%)
σ=100, coefficient=0.2	85.7 ± 1.3	35.2 ± 2.1	27.9 ± 1.3	82.3 ± 1.2
σ=200, coefficient=0.5	95.6 ± 0.8	50.1 ± 2.5	25.3 ± 1.2	95.2 ± 0.8
σ=300, coefficient=0.8	92.4 ± 0.9	65.3 ± 3.1	26.8 ± 1.2	91.4 ± 1.0
σ=200, coefficient=0.2	89.1 ± 1.1	48.5 ± 2.3	26.5 ± 1.2	88.7 ± 1.1
σ=200, coefficient=0.8	93.7 ± 0.9	52.8 ± 2.6	25.8 ± 1.2	93.6 ± 0.8
σ=150, coefficient=0.5	92.8 ± 0.9	42.7 ± 2.2	26.1 ± 1.2	90.5 ± 1.0
σ=250, coefficient=0.5	94.3 ± 0.8	57.6 ± 2.8	25.6 ± 1.2	94.1 ± 0.9

The hierarchical control module ablation analysis in Table 11 compares the proposed priority-based strategy with the unified control strategy to evaluate the effect of differentiated emergency management. Under high-priority emergency conditions, the proposed hierarchical mechanism reduces the emergency response time by 3.2 s, decreasing it from 28.5 s to 25.3 s. Meanwhile, the increase rate of regular vehicle delay is reduced from 10.1% to 4.8%, while the interference range decreases from 8 intersections to 5 intersections. The strategy pertinence score also increases by 13.5 percentage points, from 85.2 to 98.7. For medium- and low-priority emergencies, the hierarchical strategy reduces control intensity in line with event severity, limiting unnecessary disturbance to regular traffic while retaining the response needed for emergency vehicles. Compared with a unified strategy, this priority-dependent design allows control actions to vary across emergency conditions rather than applying the same intervention level to every event.

**Table 11:** Ablation table of hierarchical control module.

Grading strategy	Event Priority	Response(s)	Delay (%)	Interference range	Targetedness (%)
this method	high	25.3 ± 1.2	4.8 ± 0.5	5 ± 0.5	98.7 ± 0.5
ablation		28.5 ± 1.4	10.1 ± 0.8	8 ± 1.0	85.2 ± 1.3
this method	middle	22.4 ± 1.0	3.5 ± 0.4	2 ± 0.3	97.3 ± 0.6
ablation		25.1 ± 1.2	6.8 ± 0.6	5 ± 0.5	90.1 ± 1.1
this method	low	18.2 ± 0.9	2.1 ± 0.3	1 ± 0.2	99.2 ± 0.4
ablation		19.8 ± 1.0	3.2 ± 0.3	3 ± 0.4	94.5 ± 0.7

All quantitative entries are reported as mean ± standard deviation from three repeated experiments. The core-module ablation shows that CPC provides the largest reduction in emergency response time, while the sensitivity tests identify k = 5, M = 100, and an InfoNCE weight of 1.0 as the preferred CPC setting. For meta-learning, 10 pre-training tasks, 1000 fine-tuning samples, and α1 = 0.001 provide the strongest overall adaptation balance. The collaboration and hierarchical-control ablations further show how communication weighting and priority-dependent control affect coordination and traffic interference.

### 4.4. Results

Table 12 places the proposed framework alongside the baseline methods across the evaluated emergency scenarios. In high-priority cases, its emergency response time is 25.3 s, 15.9% below that of MARL w/o Meta. Regular-vehicle delay is 80.5 s per vehicle, corresponding to a 9.2% reduction, while the emergency success rate rises by 6.3% to 98.2%. The difference is larger under mixed emergencies: relative to the Fixed-Time strategy, response time falls by 45% and the success rate increases by 37.1 percentage points. The reported statistical test gives p < 0.05 for the performance differences across the evaluated scenarios and comparison methods. Considered together with the ablation results, these comparisons suggest that the observed gains are associated with the combined contributions of CPC-based state representation, meta-learning adaptation, inter-agent coordination, and hierarchical control rather than with any single component of the framework.

**Table 12:** Comprehensive performance results of comparative experiments.

Scene Type	Method name	Emergency response time (s)	Conventional average delay (s/vehicle)	Emergency response success rate (%)	Regular delay increase rate (%)
High priority (ambulance)	Fixed-Time	45.2 ± 3.1	120.5 ± 5.2	65.3 ± 2.1	25.7 ± 1.8
	Semi-AD	38.7 ± 2.5	105.3 ± 4.3	78.6 ± 1.7	18.2 ± 1.3
	MARL w/o CPC	35.7 ± 2.1	95.2 ± 4.2	85.6 ± 1.5	12.3 ± 1.1
	MARL w/o Meta	30.1 ± 1.5	88.7 ± 3.5	92.4 ± 1.0	7.6 ± 0.7
	this method	25.3 ± 1.2	80.5 ± 3.1	98.2 ± 0.8	4.8 ± 0.5
Medium priority (accident congestion)	Fixed-Time	40.1 ± 2.8	110.2 ± 4.8	70.5 ± 1.9	22.3 ± 1.6
	Semi-AD	33.5 ± 2.2	98.7 ± 3.9	82.1 ± 1.5	15.6 ± 1.2
	MARL w/o CPC	31.2 ± 1.9	87.6 ± 3.3	83.2 ± 1.3	9.7 ± 0.8
	MARL w/o Meta	26.8 ± 1.3	81.5 ± 3.0	91.8 ± 1.1	5.2 ± 0.6
	this method	22.4 ± 1.0	75.3 ± 2.8	97.5 ± 0.7	3.5 ± 0.4
Low priority (fire truck turning left)	Fixed-Time	35.3 ± 2.4	95.7 ± 3.6	75.8 ± 1.7	18.9 ± 1.4
	Semi-AD	29.8 ± 1.8	86.2 ± 3.2	85.4 ± 1.2	12.1 ± 1.0
	MARL w/o CPC	25.6 ± 1.4	72.3 ± 2.7	90.5 ± 1.0	6.5 ± 0.5
	MARL w/o Meta	21.3 ± 1.1	65.4 ± 2.4	95.7 ± 0.6	3.8 ± 0.4
	this method	18.2 ± 0.9	60.1 ± 2.2	99.1 ± 0.5	2.1 ± 0.3
Mixed scenario (peak + emergency)	Fixed-Time	50.7 ± 3.5	135.2 ± 5.8	58.2 ± 2.3	30.5 ± 2.1
	Semi-AD	42.3 ± 2.8	118.7 ± 4.9	70.1 ± 1.9	22.4 ± 1.6
	MARL w/o CPC	38.5 ± 2.2	102.4 ± 4.1	80.3 ± 1.4	15.2 ± 1.2
	MARL w/o Meta	33.7 ± 1.7	93.6 ± 3.7	88.5 ± 1.1	8.9 ± 0.8
	this method	27.9 ± 1.3	85.2 ± 3.2	95.3 ± 0.7	5.6 ± 0.5

Note: Values are reported as mean ± standard deviation from three repeated experiments. Differences between the proposed method and each baseline method were statistically significant (p < 0.05).

**Fig 9:**
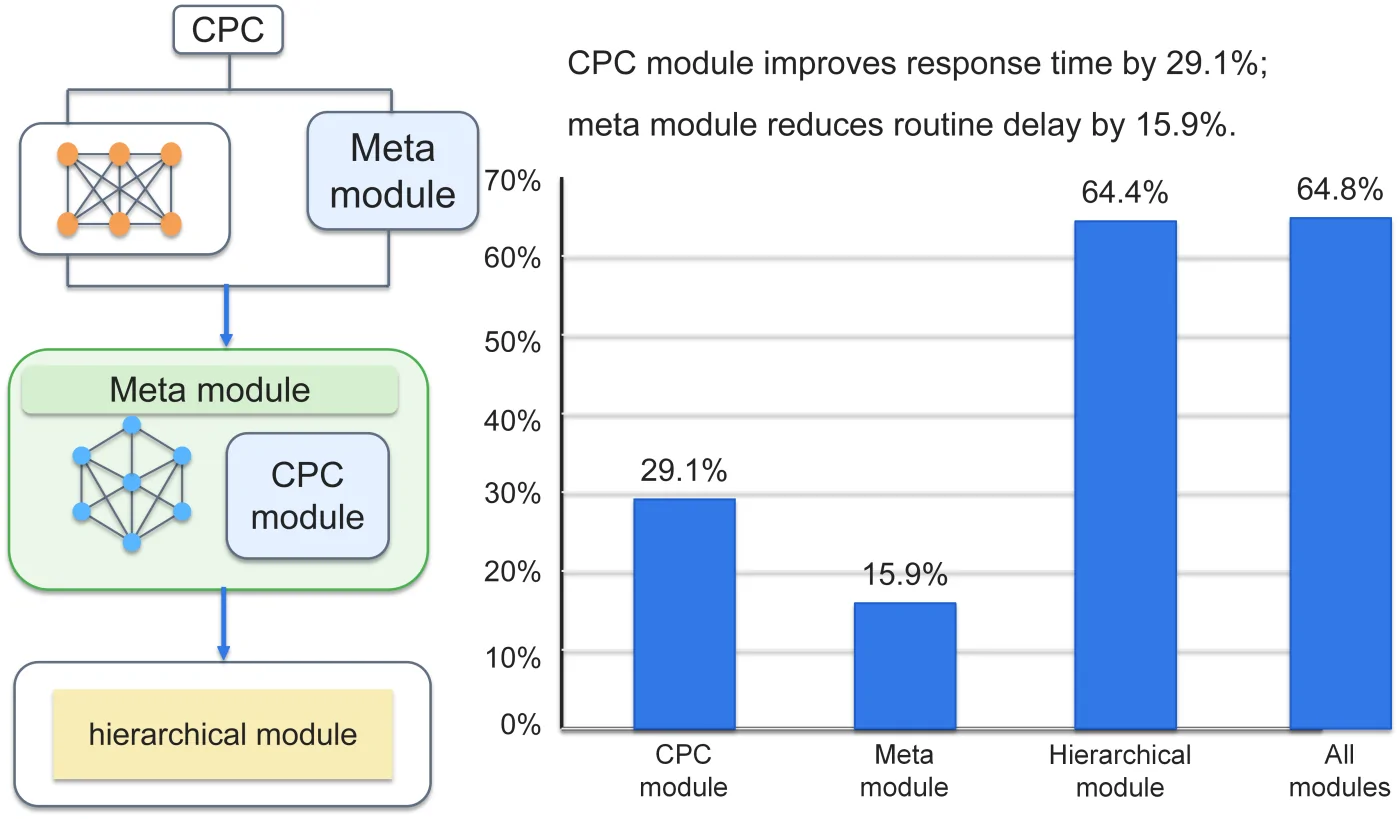
Contributions of the core modules.

Table 13 separates the performance changes associated with individual components and their combined use. Among the single-module configurations, CPC produces the largest change, reducing emergency response time by 29.1% and regular-vehicle delay by 15.4%. The result is consistent with its use of compact traffic-state representations during policy optimization. The Meta module mainly affects adaptation, with emergency response time decreasing by a further 15.9% when agents encounter previously unseen scenarios. Hierarchical control has a different effect: the delay increase rate falls by 64.4%, indicating less disruption to regular traffic when emergency priority is applied. Fig 9 shows the corresponding differences across the evaluated module configurations. When all modules are integrated, the response-time reduction contribution reaches 44.3% in the contribution analysis, indicating a non-linear combined effect rather than a simple sum of the single-module values. These results suggest that the modules complement each other through the connection between feature representation, decision adaptation, and control optimization, supporting the effectiveness of the proposed framework design.

**Table 13:** Quantification of core module contributions in ablation experiments.

Module Name	Time reduction (%)	Regular reduction (%)	Success rate improvement (%)	Traffic-interference reduction (%)
CPC feature extraction module	29.1 ± 2.3	15.4 ± 1.2	14.7 ± 1.0	61.0 ± 3.5
Meta learning module	15.9 ± 1.5	9.2 ± 0.8	6.3 ± 0.5	36.8 ± 2.1
Multi-agent collaboration module	22.5 ± 1.8	12.0 ± 1.0	9.7 ± 0.7	49.5 ± 2.8
Hierarchical control module	11.2 ± 1.1	5.5 ± 0.6	4.3 ± 0.4	64.4 ± 3.2
Collaborative contribution of all modules	44.3 ± 2.5	25.8 ± 1.5	22.8 ± 1.2	77.5 ± 3.8

Note: Positive percentages indicate improvement in the favorable direction of each metric. Response time, regular-vehicle delay, and traffic interference are expressed as reductions, whereas emergency success rate is expressed as an improvement. The all-module row represents the combined contribution observed when the modules are used together and should not be interpreted as the arithmetic sum of the single-module values.

The comparative experiments show that the proposed framework achieves improved performance across all evaluated emergency scenarios. Relative to Fixed-Time control, emergency response time is reduced by approximately 44.0%–48.4% and regular-vehicle delay by approximately 31.7%–37.2%. The emergency success rate increases by 23.3–37.1 percentage points, while the regular-delay increase rate is reduced by approximately 81.3%–88.9%. These results indicate that the proposed control strategy improves emergency passage while reducing disruption to regular traffic.

The ablation analysis separates the roles of the four main components. CPC gives the largest single reduction in emergency response time (29.1%), while multi-agent collaboration contributes 22.5% and the Meta module 15.9%. Hierarchical control has the strongest effect on interference reduction (64.4%). The combined configuration reaches a 44.3% time contribution, indicating complementary rather than purely additive effects among representation, adaptation, coordination, and priority-aware control.

### 4.5. Cross-scenario multi-objective robustness

**Fig 10:**
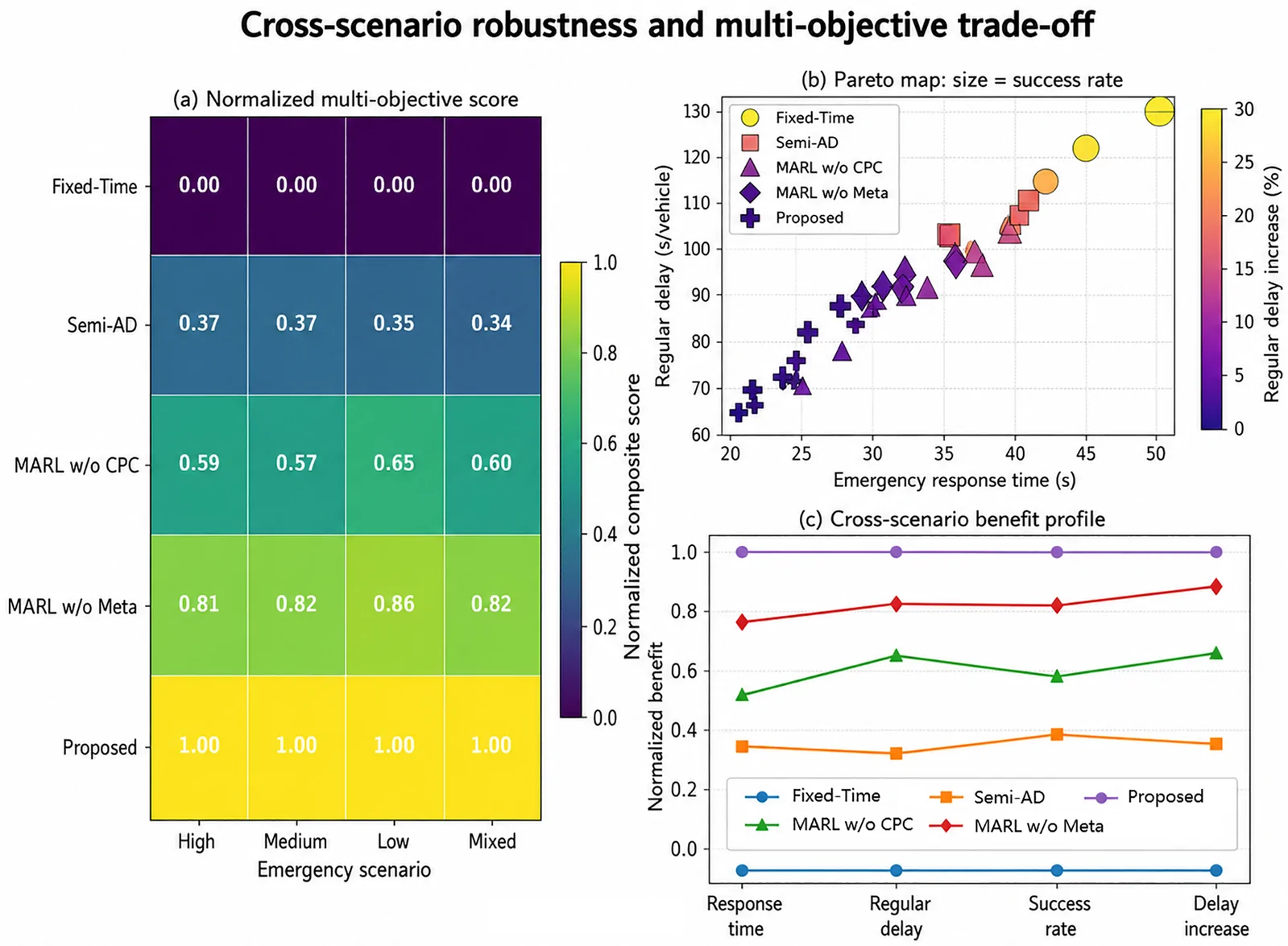
Cross-scenario robustness and multi-objective trade-off based on the results in Table 12. Marker size in panel (b) represents emergency success rate; color represents regular-delay increase.

To strengthen the result interpretation without introducing unverified observations, this analysis reuses the repeated results already reported in Table 12. Response time, regular-vehicle delay, emergency success rate, and regular-delay increase are normalized within each scenario, with lower values treated as better for the three cost metrics and higher values treated as better for success rate. Fig 10 shows that the proposed method is consistently located at the favorable boundary of the multi-objective space across high-, medium-, low-priority, and mixed emergencies. The same pattern is visible in the normalized benefit profiles, indicating that the improvement is not restricted to a single metric or event type.

### 4.6. Component interaction and contribution structure

**Fig 11:**
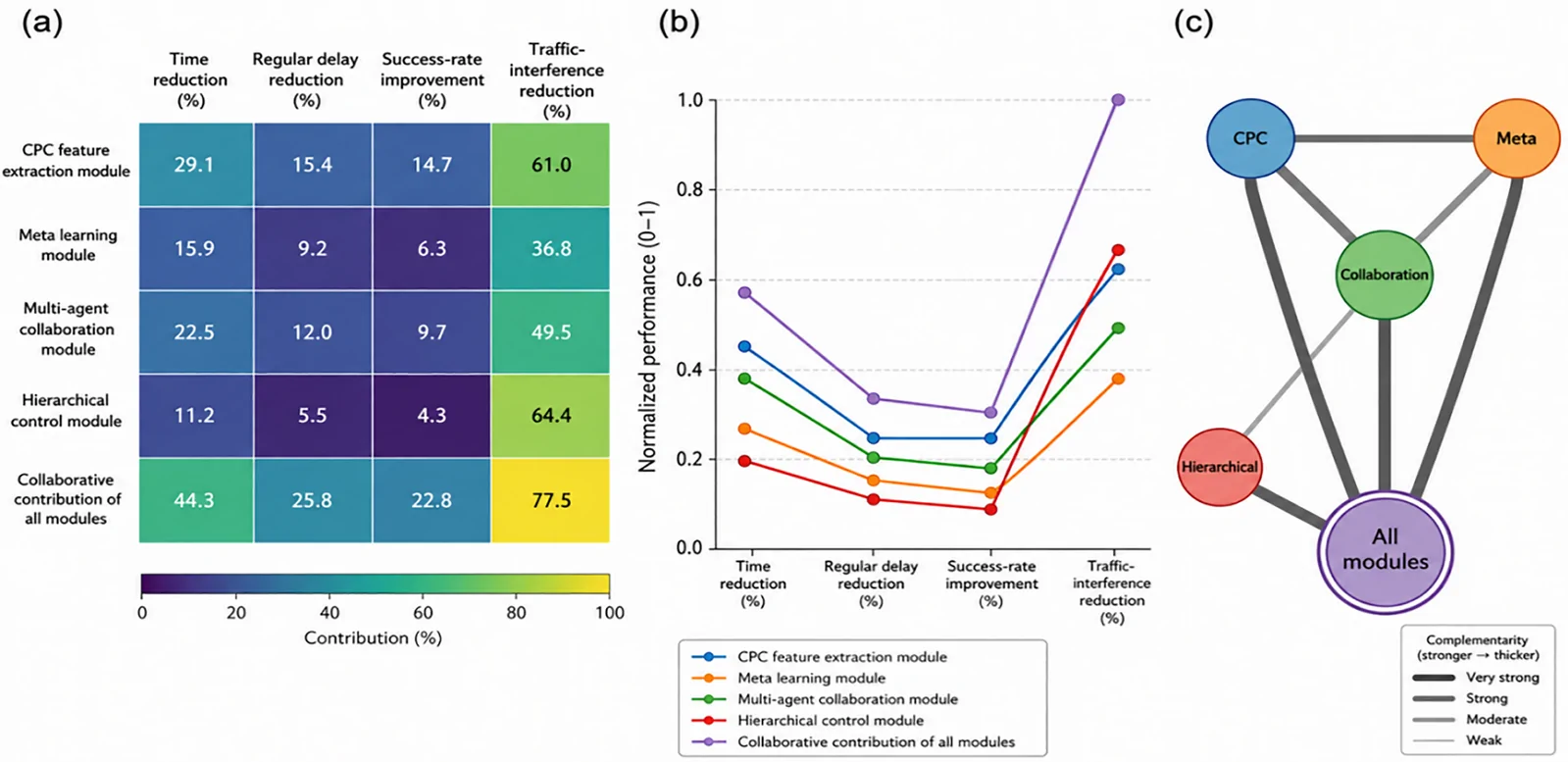
Contribution matrix, normalized module profiles, and a descriptive complementarity network derived from Table 13. Network edge strength reflects similarity between the reported contribution profiles.

The component contributions in Table 13 were further examined jointly rather than as isolated percentages. Fig 11 compares the contribution profiles of CPC, meta-learning, multi-agent collaboration, and hierarchical control. CPC is most influential for response-time reduction, whereas hierarchical control is strongest for limiting traffic interference. The all-module configuration exceeds every single module on all four reported contribution measures. This supports a complementary design in which representation learning improves the policy input, meta-learning shortens adaptation, collaboration coordinates neighboring agents, and priority-aware control limits unnecessary intervention.

### 4.7. Integrated parameter sensitivity and stability analysis

**Fig 12:**
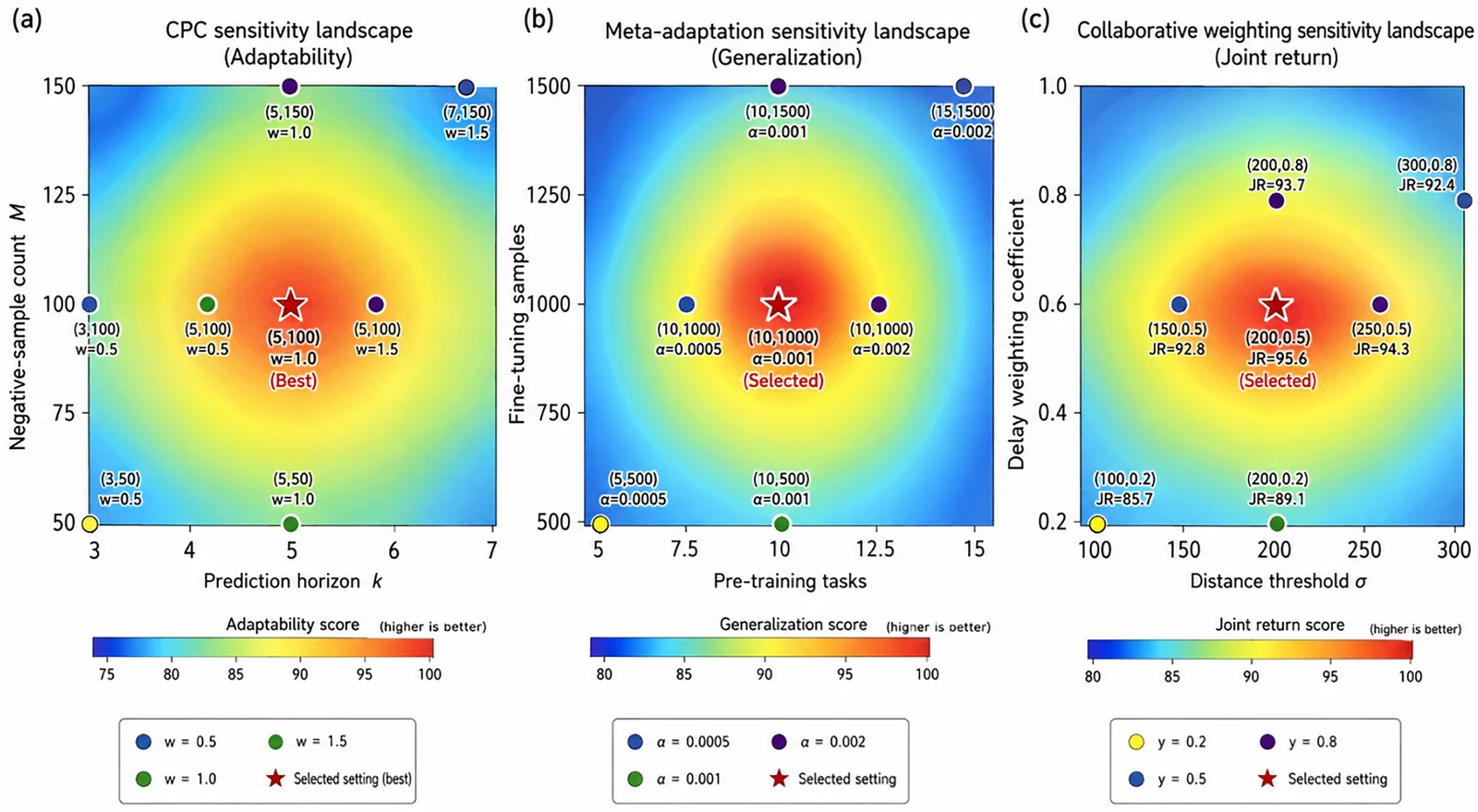
Integrated sensitivity landscapes for CPC, meta-adaptation, and collaborative weighting using the configurations reported in Tables 8–10. Surfaces are descriptive interpolations; markers are the reported settings.

Fig 12 integrates the parameter settings already reported in Tables 8–10. The CPC results favor an intermediate prediction horizon and negative-sample count, the meta-learning results favor 10 pre-training tasks with about 1000 fine-tuning samples, and the collaboration results favor a moderate distance threshold and delay weight. The markers correspond to the reported experimental configurations. The connecting surfaces are used only for visual guidance and do not represent additional measured trials. Taken together, the three landscapes show that the selected settings lie in stable regions rather than at isolated extreme points, which reduces the likelihood that the reported performance depends on one narrowly tuned parameter choice.

## 5. Discussion

### 5.1. Key findings

The experimental results show that the combination of Contrastive Predictive Coding and multi-agent meta-reinforcement learning improves emergency traffic control under the evaluated scenarios. The proposed framework records shorter emergency response times, less disturbance to regular traffic, and higher emergency handling success rates than conventional reinforcement learning-based approaches. CPC removes redundant traffic information and produces state representations that are more suitable for control decisions. Meta-learning helps agents adjust their policies when unfamiliar emergency situations occur. Multi-agent collaboration supports information exchange between intersections and improves regional coordination. Hierarchical control changes the intervention intensity according to emergency priority. This reduces unnecessary effects on regular traffic and keeps the control action consistent with the severity of the emergency.

The ablation results further show that the individual components provide different but complementary contributions to overall performance. CPC achieves the largest improvement in state representation quality, whereas meta-learning mainly improves adaptation efficiency when interaction data are limited. The results indicate that jointly considering traffic representation, adaptive policy optimization, and collaborative control can improve the robustness of emergency traffic management frameworks. The additional multi-objective and sensitivity analyses in Sections 4.5–4.7 show that this advantage is consistent across scenarios and remains stable around the selected parameter settings.

### 5.2. Research limitations

Despite the effectiveness demonstrated by the proposed framework, the current study still has several limitations. The first limitation is related to the simulation-based evaluation environment. Although the iTETRIS platform adopts traffic flow and communication parameters consistent with commonly used engineering settings, some uncertain factors in real-world deployments, such as communication fluctuations under severe weather conditions and irregular driver responses, are not fully represented. Therefore, differences may exist between simulation outcomes and practical traffic control scenarios. Another limitation concerns the fixed configuration of CPC parameters, including the prediction step size k and the number of negative samples M. These parameters are not dynamically adjusted according to changing emergency conditions, such as emergency vehicle speed variations and congestion propagation patterns, which may influence the flexibility of feature extraction. In addition, the multi-agent collaboration mechanism is currently evaluated mainly in a small-scale network with 12 intersections. Its communication efficiency, decision consistency, and computational cost in larger traffic networks, such as areas containing more than 50 intersections, require further investigation.

### 5.3. Recommendations for future research

Future research will focus on improving both the practicality and scalability of the proposed framework. Cooperation with urban traffic management departments would make it possible to collect real emergency traffic data and carry out field tests, providing a basis for adjusting the model under actual operating conditions. CPC parameters could also be updated online, with the prediction horizon and negative-sample selection changed according to the type and severity of an emergency rather than fixed in advance. For larger regional networks, a hierarchical multi-agent architecture may be considered, for example, a two-level structure with regional and intersection controllers, so that coordination does not require all agents to exchange information directly. This design could reduce communication overhead while keeping decision updates manageable. Further work may also examine federated meta-learning when traffic data cannot be centrally shared and explore the use of large-model semantic representations for recognizing emergency scenarios automatically. These directions extend the present framework toward real-world deployment, large-scale coordination, privacy-aware learning, and richer emergency perception.

## 6. Conclusions

This work develops a rapid adaptive emergency traffic control framework by integrating Contrastive Predictive Coding with multi-agent meta-reinforcement learning. The framework incorporates traffic state representation learning, adaptive policy optimization, multi-agent coordination, and hierarchical emergency control into a unified decision process. Experimental evaluations under different emergency scenarios show that the proposed method achieves improved performance compared with conventional signal control strategies and reinforcement learning-based baselines. The framework reduces emergency response time, limits the impact on regular traffic flow, improves emergency handling success rates, and enhances coordination among multiple intersections. The ablation studies further analyze the contribution of individual components and demonstrate that representation learning, meta-adaptation, collaborative decision-making, and hierarchical control jointly influence the final control performance. These results indicate that combining self-supervised representation learning with adaptive multi-agent reinforcement learning is a feasible approach for emergency traffic management in dynamic environments. The proposed framework provides a reference for future intelligent transportation systems that require efficient adaptation under changing traffic conditions.
